# Relationships between the race implicit association test and other measures of implicit and explicit social cognition

**DOI:** 10.3389/fpsyg.2023.1197298

**Published:** 2023-07-27

**Authors:** Charlotte R. Pennington, Matthew Ploszajski, Parmesh Mistry, Nicola NgOmbe, Charlotte Back, Sam Parsons, Daniel J. Shaw

**Affiliations:** ^1^School of Psychology, Aston University, Birmingham, United Kingdom; ^2^Department of Health and Social Sciences, University of the West of England, Bristol, United Kingdom; ^3^Chair of Clinical Psychology and Behavioral Neuroscience, Technische Universität Dresden, Dresden, Germany; ^4^Donders Institute for Brain, Cognition and Behavior, Radboud University Medical Center, Nijmegen, Netherlands

**Keywords:** race implicit association test, race IAT, individual differences, social cognition, implicit measures, theory

## Abstract

**Background:**

The race-based Implicit Association Test (IAT) was proposed to measure individual differences in implicit racial bias subsumed within social cognition. In recent years, researchers have debated the theoretical tenets underpinning the IAT, questioning whether performance on this task: (1) measures implicit attitudes that operate automatically outside of conscious awareness; (2) reflects individual differences in social cognition; and (3) can predict social behavior. One way to better address these research questions is to assess whether the race-IAT correlates with other implicit processes that are subsumed within social cognition.

**Aims:**

The current study assessed whether the race-IAT was related to other commonly used individual difference measures of implicit (and explicit) social cognition. Experiment 1 assessed whether dissociable patterns of performance on the race-IAT were related to measures of implicit imitative tendencies, emotion recognition and perspective taking toward White task actors, as well as explicit measures of trait and state affective empathy and racial bias. Overcoming limitations of task conceptual correspondence, Experiment 2 assessed whether these latter tasks were sensitive in detecting racial biases by using both White and Black task actors and again examined their relationships with the race-IAT.

**Method:**

In two lab-based experiments, 226 and 237 participants completed the race-IAT followed by an extensive battery of social cognition measures.

**Results:**

Across both experiments, pro-White/anti-Black bias on the race-IAT was positively related to a pro-White bias on explicit measures of positive affective empathy. However, relationships between the race-IAT and implicit imitative tendencies, perspective taking, emotion recognition, and explicit trait and negative state affective empathy were statistically equivalent.

**Conclusion:**

The race-IAT was consistently related to explicit measures of positive state affective empathy but not to other individual difference measures of implicit social cognition. These findings are discussed with regards to the theoretical underpinnings of the race-IAT as an individual difference measure of implicit social cognition, as well as alternative explanations relating to the reliability of social cognition measures and the various combinations of general-purpose (social *and* non-social) executive processes that underpin performance on these tasks.

## Introduction

The Implicit Association Test (IAT; Greenwald et al., 1998) was proposed to measure individual differences in attitudes and stereotypes that operate outside of conscious awareness but that can exert an enormous influence on behavior (Nosek et al., 2012; Greenwald and Lai, 2020). The basic premise underpinning this task is that individuals should be faster when categorizing concepts (e.g., race) with evaluative attributes (good/bad) that are associated strongly in memory. Spanning far beyond social psychology, the race- IAT has entered into policy, political, and public discourse (Spencer et al., 2016; Zestcott et al., 2016; Atewologun et al., 2018; Yen et al., 2018). In recent years, however, both the construct and predictive validity of this paradigm have come under increasing scrutiny, calling into question whether it can really measure some of the implicit cognitive processes that drive social behavior (Brownstein et al., 2019; Corneille and Hütter, 2020; Schimmack, 2021). Over the last 20 years, a vast literature has identified numerous other cognitive processes that guide our behavior in social situations, referred to collectively as social cognition; for example, our ability to recognize and empathize with other people’s emotions (see Happé et al., 2017). We hypothesized, therefore, that if the race-IAT has construct and predictive validity, performance on this measure should be correlated with other implicit measures of social cognitive processes. We start by outlining three ongoing debates regarding the IAT in further detail, all of which informed the basis of this study.

### Does the IAT measure implicit attitudes?

A common theoretical assertion is that the IAT assesses *implicit attitudes* – that is, attitudes that are automatically activated through indirect measures, and that are therefore easily concealed or inaccessible to conscious introspection when measured with self-report instruments (Greenwald et al., 1998; Nosek B. A. et al., 2007; Nosek et al., 2011; Greenwald and Lai, 2020). From this perspective, implicit and explicit measures of attitudinal constructs (e.g., racial bias) should not be highly correlated because the former identifies “introspectively unidentified traces of experience” (Greenwald and Banaji, 1995; Goedderz et al., 2023). However, some researchers have debated how the term ‘implicit’ has been used in the social cognition literature (Corneille and Hütter, 2020) and empirical evidence questions the extent to which individuals are *unaware* of their implicit attitudes measured by the IAT. For example, research has routinely shown that individuals are able to routinely predict their performance on the IAT with a high degree of accuracy (Hahn et al., 2014; Goedderz et al., 2023) and such awareness allows them to control or even fake their responses (Vanman et al., 2004; Röhner et al., 2013). Research further suggests that there may be nothing ‘implicit’ about these implicit attitudes with race-IAT scores and self-report measures mapping onto the same latent construct (Schimmack, 2021; Vianello and Bar-Anan, 2021). At current then, the construct validity of the IAT remains heavily debated, with some researchers suggesting that performance on this measure does not necessarily reflect ‘automatic’ or ‘unconscious’ attitudes (Hahn et al., 2014; Schimmack, 2021). Nevertheless, others contend that automaticity is not a unitary construct and instead includes many sub-processes, such as intention, efficiency, and cognitive control (De Houwer et al., 2009; Gawronski, 2019; Kurdi et al., 2021). As such, implicit attitudes might be available to consciousness but can still affect judgments and behaviors in a manner that bypasses awareness (Kurdi et al., 2021).

### Is the IAT an individual difference measure of social cognition?

The IAT was originally proposed to measure individual differences in social cognition (Greenwald et al., 1998). However, in the twenty years since its debut, researchers have also debated whether implicit biases do indeed reflect stable individual traits or instead malleable attitudinal concepts that vary across situations (Kurdi and Banaji, 2017; Payne et al., 2017; Rae and Greenwald, 2017; Connor and Evers, 2020). Using a ‘bias of crowds’ model, Payne et al. (2017) interpret greater stability in average IAT scores across U.S. states compared with those at the individual level to reflect the context-dependence of implicit bias (see also Hehman et al., 2019; Devos et al., 2021). This argument has been met by several rejoinders, with proponents of the individual differences perspective arguing that “implicit attitudes are characteristics of people, almost certainly more so than a property of situations” (Rae and Greenwald, 2017; pp. 297), and noting that “[whilst] the validation of the IAT is still incomplete […], the IAT is the best available candidate for measuring automatic judgment at the person level” (Vianello and Bar-Anan, 2021; pp. 415). Another aspect of Greenwald et al.’s original claim which requires further validation is whether implicit bias, as measured by the IAT, can indeed be conceptualized as a component of social cognition – a psychological construct encompassing a broad range of cognitive mechanisms involved in social information processing (Frith and Frith, 2012; Happé et al., 2017; Shaw et al., 2020). If implicit biases reflect the cognitive processes at the heart of social perception, judgment and action (Greenwald et al., 1998; Nosek et al., 2011; Kurdi and Banaji, 2017; Rae and Greenwald, 2017), then we hypothesized that performance on the (race-)IAT should be related to other socio-cognitive processes that guide how we interact with others.

### Does the IAT predict social behavior?

In addition to the construct validity of the IAT, there is also uncertainty surrounding its predictive validity – that is, the degree to which attitudes measured by this task can predict social behavior. While some studies suggest that performance on this task can predict a range of behaviors, such as racial discrimination and voting tendencies (Dovidio et al., 2002; Amodio and Devine, 2006; Greenwald et al., 2009b), meta-analytic effect sizes are small (Greenwald et al., 2009a; Oswald et al., 2013; Kurdi et al., 2019) with individual differences in IAT performance accounting for 1–8% of variance in intergroup discrimination (Kurdi and Banaji, 2017). Furthermore, re-analyses of some classic studies have failed to reproduce relationships between IAT performance and discriminatory behavior (see Blanton et al., 2009; Blanton and Mitchell, 2011; Schimmack, 2021). In this light, some researchers suggest that the predictive validity of the IAT has been overstated (Oswald et al., 2013, 2015) and may not achieve its intended purpose of overcoming the limitations of self-reports (Meissner et al., 2019). However, it might be unfair to expect strong correlations between implicit attitudes and overt social behaviors when the latter are equally susceptible to self-presentational motives and social desirability as self-report indices. Indeed, researchers suggest that it is rare for any implicit measure to correlate strongly with behavioral outcomes measured explicitly (Campbell and Fiske, 1959; Brownstein et al., 2020). A more accurate assessment of the IAT’s construct and predictive validity therefore requires research to investigate whether individual differences on this task are related to other implicit measures of social cognition that are less accessible or amenable to conscious control (see also Nosek et al., 2011; Gawronski, 2019).

Social cognition is often conceptualized as a hierarchical structure of numerous distinct yet inter-related cognitive processes involved in processing socially relevant information that operate largely beyond conscious awareness (Frith and Frith, 2012; Happé et al., 2017; Shaw et al., 2020). Various measures have been developed to assess each constituent social cognitive process. To assess if and how implicit racial bias, as measured by the race-IAT, is related to these other social cognitive processes, we constructed a test battery comprising tasks designed specifically to measure a selection of them. In the sections that follow, we outline our rationale for their inclusion.

One fundamental socio-cognitive process is imitation – humans exhibit an involuntary tendency to imitate one another during social interaction, which serves to enhance rapport and affiliation (van Baaren et al., 2009; Heyes, 2011; Chartrand and Lakin, 2013). We do not imitate everyone equally, however, with both behavioral and neuroscientific investigations revealing that we are more likely to imitate individuals with perceived similarities to ourselves (Bourgeois and Hess, 2008; Gleibs et al., 2016; Rauchbauer et al., 2018), including individuals of our own race (Cazzato et al., 2018). Importantly, this appears to be associated negatively with intergroup biases (Yabar et al., 2006; Gutsell and Inzlicht, 2010; Cazzato et al., 2018; Darda et al., 2020), suggesting that social attitudes are linked in some way to imitative tendencies (see also Leighton et al., 2010).

These imitative tendencies appear to be related also to the socio-cognitive processes supporting low-level (level-1) *visual perspective taking* (VPT) – that is, the ability to detach ourselves from our own viewpoint in order to infer what lies within someone else’s line of sight (Michelon and Zacks, 2006; Samson et al., 2010). A large corpus of research has shown that VPT occurs automatically without top-down control and is susceptible to both ego- and alter-centric misattributions (Samson et al., 2010; Surtees and Apperly, 2012; Furlanetto et al., 2016). Studies assessing the impact of shared group membership on this ability have reported somewhat contrasting findings, however: Simpson and Todd (2017) found that egocentric misattribution impaired VPT for in-group members, while Schneider et al. (2018) found this same effect for the out-group. Interestingly, Schneider et al. also found evidence for an implicit in-group bias that may have modulated this effect, and several findings indicate that implicit racial bias is reduced by improving perspective-taking ability (Todd et al., 2011, 2012; Berthold et al., 2013; Peck et al., 2013). This is suggestive of a relationship between VPT and implicit racial bias.

Perspective-taking ability has also been shown to be related to the socio-cognitive processes involved in *affective empathy* (Lamm et al., 2007, 2008; Mattan et al., 2016) – the vicarious experience of another person’s affective state (Shamay-Tsoory and Lamm, 2018). Research indicates that empathic processes can be influenced by the social groups to which people belong (Cikara et al., 2011; Cikara and Van Bavel, 2014; Zaki, 2014), with individuals failing to empathize with out-group members and showing attenuated responses to their suffering (Xu et al., 2009; Chiao and Mathur, 2010). This is further supported by research that reveals relationships between affective empathy and implicit intergroup bias (Avenanti et al., 2010; Forgiarini et al., 2011; Cazzato et al., 2018; Fabi and Leuthold, 2018). This may be explained by the in-group empathy hypothesis, which posits that people show exaggerated affective responses to both positive and negative depictions of in- relative to out-group members (Brown et al., 2006; Stürmer et al., 2006).

In order for us to experience empathy, we must be able to accurately detect the emotions expressed by others – a social cognitive process referred to as *emotion recognition* (Besel and Yuille, 2010; Coll et al., 2017). Again, a wealth of research demonstrates an in-group advantage in emotion recognition; people are better able to detect the emotions of similar relative to dissimilar others (Elfenbein and Ambady, 2002; Young and Hugenberg, 2010), and this appears to be related to implicit racial bias (Gutsell and Inzlicht, 2012). This is supported further by interventions that appear to improve intergroup relations by enhancing empathy toward outgroup members (Batson and Ahmad, 2009; Inzlicht et al., 2012; Whitford and Emerson, 2019). Taken together, this body of research suggests that the myriad of socio-cognitive processes that guide how we interact with others is modulated by our implicit attitudes toward social groups.

## Research overview

In a bid to address the unresolved questions surrounding the construct and predictive validity of the race-IAT, the current study assessed whether individual differences in race-IAT performance are related to other implicit (and explicit) measures of social cognition. To achieve this, alongside the race-IAT, we selected indirect measures from the broader literature on social cognition designed to assess imitative tendencies, emotion recognition, and visual perspective taking (see Frith and Frith, 2007, 2012; Bukowski and Samson, 2017; Happé et al., 2017; Shaw et al., 2020), as well as explicit self-report measures of trait and state affective empathy and perspective taking. In Experiment 1 we predicted that participants expressing stronger relative to weaker pro-White/anti-Black implicit bias on the race-IAT to also show biased responding to White actors on our other measures of social cognition; specifically, greater imitative tendencies, superior VPT, more efficient emotion recognition and heightened affective empathy. We also predicted that such relationships would be stronger between the race-IAT and our other implicit relative to explicit measures of social cognition. In Experiment 2 (*preregistered*), we then overcame issues of conceptual correspondence between the race-IAT and our other socio-cognitive measures in Experiment 1 by also manipulating the race of the task actor (White and Black) in the latter tasks. In this second experiment, we first assessed whether our measures of social cognition were indeed sensitive enough to detect racial biases before assessing their relationship with the race-IAT. We predicted that White and Black participants would exhibit greater imitative tendencies, more proficient VPT and emotion recognition, and greater affective empathy toward own-race relative to other-race task actors. In turn, we predicted that this preferential social information processing of White or Black actors would be related to implicit racial bias; for example, higher expressions of pro-White/anti-Black bias on the race-IAT would be related to greater biases toward White relative to Black actors on the other tasks of social cognition.

## Experiment 1

### Materials and methods

#### Data availability and transparency statement

The materials, code, and anonymized data are publicly available on the Open Science Framework.^1^ In the sections that follow, we report all measures, manipulations, and exclusions.

#### Participants

Recommendations suggest that a sample size of 193 participants is required to detect two-tailed correlations of *r* = 0.20 with 80% power and *α* = 0.05 [a ‘typical’ correlation as proposed by Gignac and Szodorai (2016)], and sample sizes should approach 250 for stable correlation estimates (Schönbrodt and Perugini, 2013). We therefore aimed to recruit a minimum of 200 participants and continued with data collection to the end of the university term to allow psychology students to acquire course credits. A total of 241 students were recruited from two UK universities, but 9 were removed due to incomplete data and 6 having reported their race as ‘other’. The final sample comprised 226 participants (200 females, *M*_AGE_ = 20.25, *SD* = 3.37) of whom 150 identified as White, 22 Black, and 54 Asian. Sensitivity power analyses conducted with the ‘pwr’ package in *R*-Studio [v.1.3–0; (Champely, 2020)] indicated that this sample had 80% power to detect two-tailed correlations of *r* > 0.18. This research was approved by the ethical review committee at both universities (References: HAS.18.07.201 and #1384) and was carried out in accordance with the Declaration of Helsinki. All participants provided informed written consent.

#### Measures and procedure

**Figure 1:**
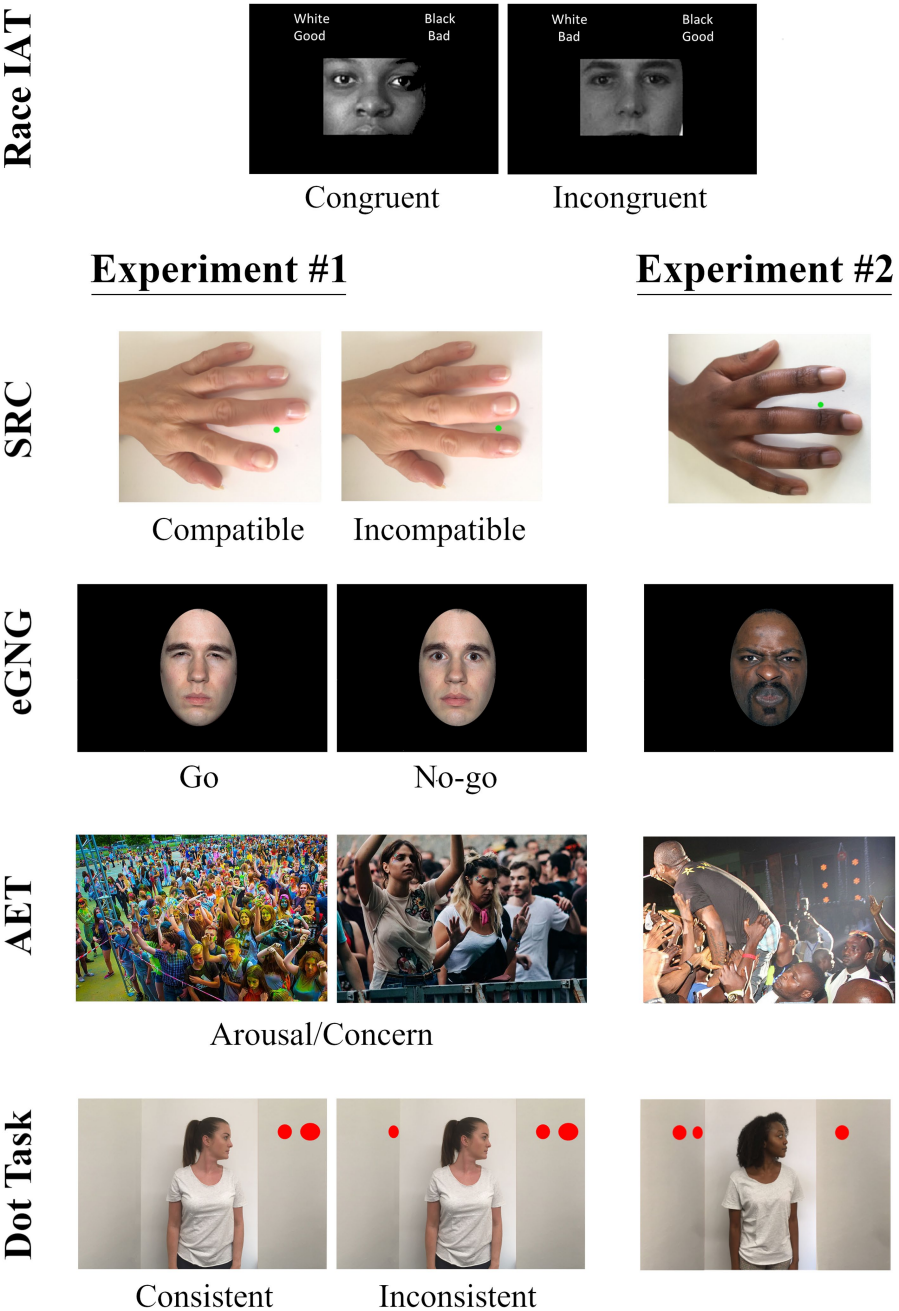
Procedural overview of experimental tasks employed in Experiment 1 (White actor only) and Experiment 2 (White and Black actors). Stimuli from the IAT, SRC, eGNG, and VPT are publicly available for research use and represent actual exemplars. Stimuli from the AET were selected from the IAPS and are not presented here due to copyright restrictions; for this figure we have replaced these with publicly available alternatives. Written informed consent was obtained from the individuals for the publication of any potentially identifiable images included in this article.

A battery of experimental tasks and self-report instruments was administered *via* the Cogent (v1.31) toolbox in MATLAB (The Mathsworks Inc, 2017). These were presented in the fixed order that they appear below, which is preferable when correlating variables to assess individual differences (Hedge et al., 2018). Task reliability was estimated using the split-half method for behavioral tasks with 5,000 random permutations (Parsons et al., 2019; Parsons, 2021) and omega total (Ω) for self-report questionnaires (Revelle and Condon, 2019). Figure 1 provides an overview of the tasks in both Experiment 1 and 2, and additional methodological details are presented in the Supplementary material.

#### Implicit racial bias

Implicit racial bias was measured using the race-IAT (Greenwald et al., 1998) with the stimuli selected from the Project Implicit website. This dual-categorization task requires participants to classify White and Black faces and/or positive and negative attributes as fast as they can by pressing one of two assigned computer keys. The IAT comprised seven blocks, including five blocks of 20 practice trials and two critical blocks of 40 trials. In one critical block, participants used the same response key to categorize White faces and positive words (White/Good), and Black faces and negative words (Black/Bad). On the alternate critical block, these categorizations were reversed. In line with the IAT literature, we refer to these herein as Congruent and Incongruent blocks, respectively, which were presented in a counterbalanced order. Within all blocks, the inter-trial interval was 1,000 ms, consisting of a white fixation cross, and incorrect categorizations were indicated by a red cross that was displayed until a correct response was given. IAT *D*-scores were calculated according to the conventional revised scoring algorithm (Greenwald et al., 2003), and the reliability of this measure was acceptable (split-half 0.88; [95% CI 0.72, 0.82]). Given that the IAT *D*-score is related monotonically to Cohen’s *d*, we classified participants’ IAT scores as expressions of weak (*d* = 0.20), moderate (*d* = 0.50) or strong (*d* = 0.80) implicit racial bias (Nosek et al., 2002; Nosek and Sriram, 2013). Positive scores indicate greater pro-White/anti-Black bias, and negative scores indicate greater pro-Black/Anti-White bias.

#### Imitative tendencies

Imitative tendencies were measured using a Stimulus–Response Compatibility task (SRC; Brass et al., 2001). This task requires participants to execute finger-lifting actions in response to a colored dot (imperative stimulus) while observing task-irrelevant finger movements performed by a White actor’s hand (stimulus hand). The degree to which participants are faster to execute the response signaled by the imperative stimulus when they observe simultaneous compatible (matching) relative to incompatible (opposing) finger movements is taken as an index of their automatic tendency to imitate the actions of others (also referred to as automatic imitation; see Heyes, 2011; Cracco et al., 2018).

The SRC task comprised one block of 72 randomized trials depicting a White actor’s left hand. This hand was rotated 90° counter-clockwise from the participant’s perspective to avoid confounding spatial compatibility effects (Bertenthal et al., 2006; Boyer et al., 2012). In each block, there were 24 Compatible trials in which the stimulus hand performed the same finger extension that was signaled by the imperative stimulus; 24 Incompatible trials in which the stimulus hand extended the opposite finger to that signaled by the imperative stimulus; and 24 Baseline trials in which the imperative stimulus was shown but the stimulus hand did not perform a finger extension. After removal of responses greater than 1,000 ms, we subtracted mean RTs on Compatible from Incompatible trials. However, the reliability of this difference score was low (split-half 0.16; [95% CI −0.01, 0.32]; see “General Discussion”). Higher values correspond to greater imitative tendencies.

#### Emotion recognition

Emotion recognition was measured using an emotional Go/No-Go task (eGNG; Tottenham et al., 2011). The stimuli comprised an image set of White actors expressing various emotions selected from the NimStim database (Tottenham et al., 2009). The eGNG task comprised six blocks of 40 trials; each trial started with a fixation-cross presented for 1,000–2,000 ms, followed by a face stimulus presented for 500 ms. During three emotional Go blocks, participants were required to press the space bar as quickly as possible whenever a specific emotion was presented (Go trials) and inhibit this response to neutral expressions (No-go trials). Each emotion (happy, sad, angry) was presented in a separate block. During three non-emotional Go blocks, these instructions were reversed. In each block, Go trials occurred frequently (70%) to evoke a prepotent tendency to respond. Both blocks and the order of Go and No-go trials were pseudorandomized. As a measure of emotion recognition, we computed the hit rate across all three emotional blocks – that is, the proportion of Go trials in which the participant responded correctly to an emotional expression. The reliability of this measure was acceptable (split-half 0.70; [95% CI 0.63, 0.75]). Higher values correspond to better emotion recognition.

#### Trait empathy

Some studies demonstrate a relationship between the (race)IAT and explicit behavioral outcomes (Greenwald et al., 2009a), so we also acquired self-report measures of trait empathy using the Interpersonal Reactivity Index (IRI; Davis, 1983). This 28-item questionnaire consists of four 7-item sub-scales, but the current study focused specifically on those indexing Perspective Taking (PT), Empathic Concern (EC), and Personal Distress (PD). Participants responded to statements (e.g., “*I often have tender, concerned feelings for people less fortunate than me*”) on a 5-point Likert scale (1 = Does not describe me well, 5 = Describes me very well). Responses to items for each sub-scale were summed and all had acceptable reliability (PT Ω = 0.78, EC = 0.78, PD = 0.67). Higher scores correspond to higher self-reported PT, EC, and PD.

#### Visual perspective taking

We measured level-1 visual perspective taking (VPT) with the Dot task (Samson et al., 2010). The stimuli comprised a lateral view of a room with a White actor standing in the center, looking toward the left- or right-hand wall. The sex of the actor was matched to the participant’s own gender and, in line with Langton (2018), we developed stimuli to depict human task actors rather than computerized avatars. On experimental trials, one to three red discs were presented on one or both walls. During 50% of these trials, the actor’s gaze meant that s/he saw the same number of discs as the participant (“Consistent”); for the remainder, the number of discs visible to the actor was different to that seen by the participant (“Inconsistent”).

Each trial began with a fixation cross presented for 750 ms. After 500 ms, the word ‘Self’ or ‘Other’ was presented for 750 ms, informing the participant to take their own or the actor’s perspective. After another 500 ms, a digit between one and three was presented for 750 ms. Participants were instructed to indicate within 2,000 ms whether this digit “matched” or “mismatched” the number of dots that could be seen from the instructed perspective. In each of four blocks there were 48 experimental and 4 filler trials, the former divided equally among the factorial combination of Perspective (Self vs. Other), Consistency (Consistent vs. Inconsistent) and Trial Type (Matching vs. Mismatching). The order of trials was pseudorandomized, and blocks counterbalanced across participants. As per Bukowski and Samson (2017), we averaged participants’ reaction times (ms) across Other-Inconsistent match trials to create a single-dimension index of perspective taking. The reliability of this measure was acceptable (split-half 0.84; [95% CI 0.80, 0.87]). Higher values (slower responses) correspond to poorer perspective-taking performance.

#### State affective empathy

The State Affective Empathy task (SAE; Brown et al., 2006) was employed as an explicit measure of state empathic concern and arousal, which has been shown to correlate with the IRI (Dziobek et al., 2008). Thirty-eight photographs were selected from the International Affective Picture System (IAPS; Lang et al., 2008). Normative valence ratings were then used to create a stimulus set depicting White people expressing negative (*n* = 13^2^), positive (*n* = 14) and neutral (*n* = 10, filler) emotions in various contexts. For each stimulus, participants were presented with two questions designed to capture affective empathy (Dziobek et al., 2008), “*How concerned are you for the person in the image?*” and “*How aroused (i.e., calmed or excited) are you by the image?*” responding to each using a Self-Assessment Manikin on a 9-point scale (1 = Not very, 9 = Very much). Measures of empathic concern were derived by summing responses to the first question for positive (*Ω = 0*.89) and negative images (*Ω* = 0.87), and arousal was measured by summing responses to the second question for positive (*Ω = 0.*91) and negative images (*Ω* = 0.93). This resulted in four dependent variables, referred to herein as Concern*_POS_*, Concern*_NEG_*, Arousal*_POS_* and Arousal*_NEG_*. Higher values correspond to greater affective empathy on each of these subscales.

#### Explicit racial bias

Finally, in order to examine whether the race-IAT predicted social cognition to a greater extent than explicit racial bias, we also included three self-report questions of explicit racial bias (Greenwald et al., 2009b). Participants first responded to two questions on a 10-point scale (0 = Very cold, 9 = Very warm): “*How do you feel White people?*” and “*How do you feel towards Black people?*.” Subsequently, they responded to a third: “*Do you have a preference for Black or White people?*” on a 3-point scale (1 = Strongly prefer Black people, 3 = Strongly prefer White people). Subtracting responses to the second from the first question created one self-report index, referred to herein as relative warmth toward White people (“Warmth*_W_”*). Responses to the third question provide a separate measure referred to as relative preference toward White people (“Preference*_W_”*). Higher scores on Warmth*_W_* and values greater than 2 on Preference*_W_* correspond to a Pro-White/anti-Black bias.

#### Analytic strategy

All analyses were conducted using SPSS (v.26; IBM Corp, 2019). Participants achieving 50% accuracy or less on accuracy-based tasks (SRC task, *n* = 13; Dot task, *n* = 14) were excluded from analyses of those specific tasks. Performance checks indicated that each task was performed as expected: IAT scores differed significantly from zero showing a pro-White/anti-Black bias (*M* = 0.32, *SD* = 0.36, *p* < 0.001) and this was positively related to explicit indices of Warmth*_W_* and Preference*_W_*; RTs were greater on Incompatible relative to Compatible trials of the SRC task (*p* < 0.001); on Inconsistent compared with Consistent trials of the Dot task (*p* < 0.001); and affective empathy was greater for negative relative to positive valence images on the SAE (*p* < 0.001).

To first assess individual differences in race-IAT performance, we conducted a two-step cluster analysis. Cluster analysis is a data-driven approach to behavioral classification which is used to uncover homogeneous profiles of individual differences in a particular construct in order to maximally predict differences among them and other constructs (Chiarello et al., 2012; Rocca et al., 2016; Bukowski and Samson, 2017; Ouimet et al., 2017). This therefore allowed us to identify variability in race-IAT performance based on participant’s IAT *D-scores* (e.g., weak, moderate, or strong Pro/Anti-Black/White bias) and assess differences in the other socio-cognitive measures based on these clustered scores. In line with best practice guidelines (Clatworthy et al., 2005), Bayesian Information Criterion (Schwarz, 1978) was employed to determine the best-fitting model and the log-likelihood assessed the probability that each observation belonged to a given cluster assignment. In order to validate this analysis, we then performed a chi-square test to assess whether the identified race-IAT clusters significantly differed based on participant’s self-reported race and their explicit racial bias. This was informed by previous research which has consistently demonstrated that individuals from Western cultures typically show a pro-White/anti-Black bias on the IAT, but the magnitude of this differs according to their own race (Nosek et al., 2002; Nosek B. et al., 2007; Lai et al., 2014; Rae and Greenwald, 2017). After this initial validation, we then conducted a series of one-way ANOVAs to assess whether these dissociable clusters of implicit racial bias differed on the other measures of social cognition. Bonferroni corrections were applied to pairwise comparisons.

Finally, we conducted bivariate correlations between implicit and explicit racial bias and our other measures of implicit and explicit social cognition, with any significant relationships followed by stepwise linear regressions to determine whether IAT scores explained any incremental predictive validity over explicit bias scores in predicting social cognition. In the correlation matrix, coefficients are presented both with and without the attenuation-correction formula (Spearman, 1904), which corrects for the influence of task measurement error. Note that this formula also corrects the confidence intervals around the coefficient, so there is no change to statistical significance. For the results that follow, equivalence tests (Lakens, 2017) are reported to determine whether non-significant findings are statistically equivalent or inconclusive (see Supplementary material). We set our smallest effect size of interest (SESOI) for correlation analyses at *r* = 0.20, in line with our *a priori* sample size justification, and for tests of mean differences we converted this to Cohen’s *d* = 0.50 (*d_z_* = 0.35).

### Results

**Figure 2:**
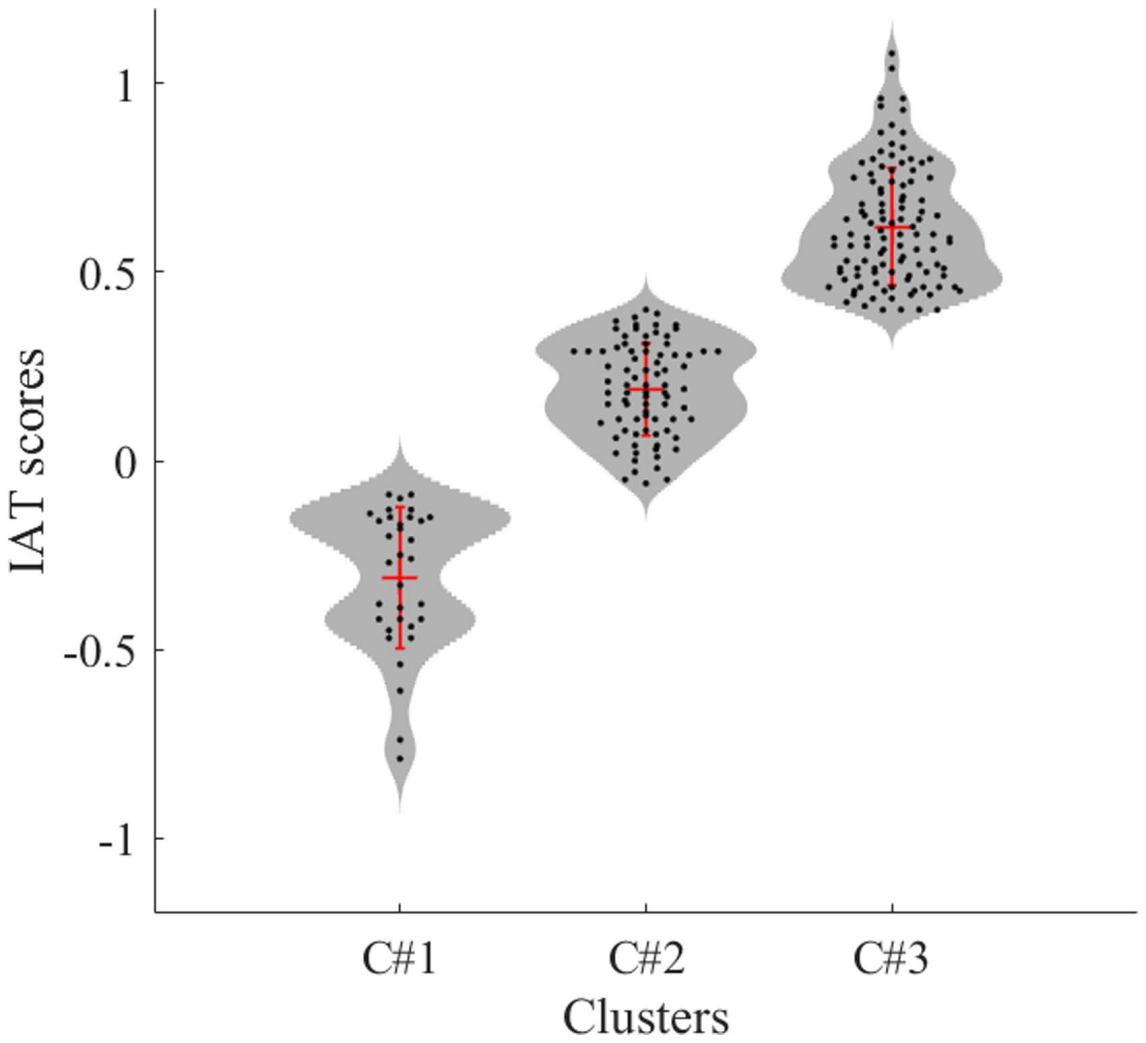
Violin plots illustrating the frequency distributions of IAT scores within each cluster. The red cross indicates cluster means and standard deviations (horizontal and vertical lines, respectively).

Cluster analysis performed on race-IAT scores revealed an optimal three-cluster solution with good model fit (0.70). Participants in cluster 1 (C#1; *n* = 33) were classified as expressing primarily weak pro-Black/Anti-White bias (*M* = −0.31, *SD* = 0.19, range = ˗0.79, ˗0.09), while those in cluster 2 (C#2; *n* = 84) were classified as expressing primarily weak pro-White/anti-Black bias (*M* = 0.19, *SD* = 0.12, range ˗ 0.06, 0.40). Participants in cluster 3 (C#3; *n* = 109) were classified as expressing moderate pro-White/anti-Black bias (*M* = 0.62, *SD* = 0.16, range 0.40, 1.08). To verify cluster stability, we repeated this analysis in a split half of the sample, revealing the same cluster solution and a similar pattern of means. Figure 2 presents the frequency distributions for each cluster.

A chi-square test indicated that these clusters of race-IAT scores varied systematically both between *and* within participant race: 93% of White participants were classified as expressing either moderate (C#3; 57%) or weak pro-White/anti-Black bias (C#2; 36%); 78% of Black participants were classified as expressing either weak pro-Black/anti-White (C#1; 46%) or weak pro-White/anti-Black bias (C#2; 32%); and 76% of Asian participants were classified as expressing weak (C#2, 43%) or moderate pro-White/anti-Black bias (C#3, 33%; *χ*[4, 226] = 32.81, *p* < 0.001, *ϕ* = 0.27). There was also a significant main effect of cluster membership on indices of explicit Warmth*_W_*, *F*(2, 223) = 10.03, *p* < 0.001, *η_p_^2^* = 0.08. Participants expressing weak pro-Black/anti-White bias (C#1) reported less warmth toward White people compared with those expressing weak pro-White/anti-Black bias (C#2; *p* = 0.03, *d* = 0.44). Furthermore, this effect was larger when comparing participants in C#1 with those expressing moderate pro-White/anti-Black bias who reported relative neutrality (C#3; *p* < 0.001, *d* = 1.07). The observed effect size was inconclusive between C#2 and C#3 (*p* = 0.07, *d* = 0.35). There was also a significant main effect of IAT cluster on Preference*_W_*, *F*(2, 223) = 11.69, *p* < 0.001, *η_p_^2^* = 0.10. Participants in C#1 reported a slight preference for Black over White people compared with those in C#2 who reported very little preference (*p* = 0.009, *d* = 0.59). This effect was again larger for the comparison between C#1 and C#3, with the latter reporting a slight preference for White over Black people (*p* < 0.009, *d* = 0.91). The observed effect size was again inconclusive between C#2 and C#3 (*p* = 0.07, *d* = 0.43).

When assessing differences between race-IAT clusters and our other measures of social cognition, there was a significant main effect on Arousal*_POS_*, *F*(2, 223) = 7.45, *p* < 0.001, *η_p_^2^* = 0.06. Participants in C#3 expressing moderate pro-White/anti-Black bias reported higher explicit positive arousal for White task actors relative to those expressing weak pro-White/anti-Black (C#2; *p* = 0.009, *d =* 0.43) and weak pro-Black/anti-White implicit bias (C#1; *p* = 0.004, *d* = 0.65). The observed effect size was inconclusive between C#1 and C#2 (*p* = 0.94, *d* = −0.25). Against predictions, the observed effect sizes between IAT clusters were statistically equivalent for imitative tendencies, emotion recognition, EC, visual perspective taking, and Arousal*_NEG_* (*d*s < 0.17, all *p* > 0.67). The observed effect sizes between clusters on PT, PD, Concern*_POS_* and Concern*_NEG_* were either inconclusive or statistically equivalent. Table 1 presents the cluster characteristics as well as the descriptive statistics for all measures.

**Table 1:** Characteristics of IAT cluster membership.

	Cluster Membership			Total sample
	C#1	C#2	C#3	
	Weak pro-Black/anti-White	Weak pro-White/anti-Black	Moderate pro-White/anti-Black	
Demographics				
*N* (% total)	33 (14.6%)	84 (37.2%)	109 (48.2%)	226
White %	6.7%	36.0%	57.3%	66.4%
Black %	45.5%	31.8%	22.7%	9.7%
Asian %	24.1%	42.6%	33.3%	23.9%
Female %	84.8%	92.9%	86.2%	88.5%

Measures	Mean (SD)			
IAT score	−0.31 (0.19)	0.19 (0.12)	0.62 (0.16)	0.32 (0.36)
Warmth*_W_*	−0.1.09 (1.89)^a,b^	−0.31 (1.75)^a^	0.17 (0.94)^b^	−0.19 (1.50)
Preference*_W_*	1.73 (0.45)^c,d^	1.95 (0.34)^c^	2.07 (0.35)^d^	1.98 (0.38)
Imitation	25.05 (47.81)	27.14 (42.77)	20.66 (39.70)	23.70 (41.91)
eRec	0.88 (0.08)	0.87 (0.07)	0.87 (0.06)	0.87 (0.07)
EC	21.42 (4.44)	20.98 (4.87)	20.92 (4.41)	21.01 (4.57)
PT	18.39 (5.31)	18.21 (4.46)	17.86 (4.75)	18.07 (4.71)
PD	13.42 (4.12)	13.71 (4.23)	14.23 (4.35)	13.92 (4.26)
VPT	863.93 (153.36)	863.42 (197.09)	886.18 (183.88)	874.23 (184.37)
Concern*_POS_*	20.73 (9.84)	22.89 (12.09)	25.13 (12.39)	23.65 (11.99)
Concern*_NEG_*	69.52 (21.05)	73.99 (18.22)	75.62 (19.25)	74.12 (19.17)
Arousal*_POS_*	30.64 (15.51)^e^	34.70 (16.70)^f^	43.25 (22.44)^e,f^	38.23 (20.10)
Arousal*_NEG_*	45.88 (24.38)	45.54 (22.35)	49.47 (25.46)	47.48 (24.16)

Common sub-scripts represent significant differences between cluster membership (*p* < 0.05). IAT, Implicit Association Test; Warmth_W_, explicit White warmth; Preference_W_, explicit White preference; Imitation, imitative tendencies; eRec, emotion recognition; EC, self-reported empathic concern; PT, perspective taking; PD, personal distress [sub-scales of trait empathy]; VPT, level-1 visual perspective taking; Concern_POS_ and Concern_NEG_, empathic concern toward positive and negative valence images; Arousal_POS,_ Arousal_NEG_, arousal toward positive and negative valence images [subscales of state affective empathy].

Correlation analyses indicated that both race-IAT scores and Warmth*_W_* showed a positive relationship with explicit Concern*_POS_* and Arousal*_POS._* Follow-up regression analyses indicated that race-IAT scores and Warmth*_W_* together explained 3.6% of the variance in Concern*_POS_, F*(2, 223) = 4.16, *p* = 0.017, but the race-IAT did not explain any incremental predictive validity (*p* = 0.11, *R*^2^Δ = 0.011). Race-IAT scores and Warmth*_W_* also explained 6.4% of variance in Arousal*_POS_, F*(2, 223) = 7.57, *p* = 0.001, with the IAT explaining an additional 3.9% of variance, *p = 0*.003. Relationships were statistically equivalent between the race-IAT and imitative tendencies, emotion recognition, visual perspective taking, explicit trait empathy (EC, PT, PD) and Arousal*_NEG,_* but inconclusive between race-IAT scores and Concern*_NEG_*. Table 2 presents the correlation matrix.

**Table 2:** Correlation matrix showing relationships between implicit and explicit racial bias and social cognition in Experiment 1.

	IAT	Warmth*_W_*	Preference*_W_*	Imitation	eRec	EC	PT	PD	VPT	Concern *POS*	Concern *NEG*	Arousal *POS*	Arousal *NEG*
IAT	0.88	0.30	0.26	−0.03	−0.05	−0.01	−0.05	0.04	0.08	0.17	0.15	0.26	0.09
Warmth*_W_*	0.28***	NA	0.55	0	−0.1	−0.24	−0.05	0.09	−0.13	0.17	0	0.17	−0.04
Preference*_W_*	0.24***	0.55***	NA	−0.12	−0.04	−0.23	−0.07	0.07	−0.04	0.14	0	0.12	0.03
Imitation	−0.01	0.001	−0.05	0.16	0.18	−0.11	−0.06	−0.03	−0.03	−0.16	−0.08	−0.1	−0.05
eRec	−0.04	−0.08	−0.03	0.06	0.70	0.18	0.05	0.07	−0.20	0.01	0.13	0.04	0.17
EC	−0.006	−0.21**	−0.20**	−0.04	0.13*	0.78	0.74	0.36	0.16	−0.1	0.42	−0.08	0.29
PT	−0.04	−0.04	−0.06	−0.02	0.04	58***	0.78	0.03	0.05	−0.06	0.28	−0.05	0.25
PD	0.03	0.07	0.06	−0.01	0.05	0.26***	0.02	0.67	0.12	0.12	0.24	−0.03	0.03
VPT	0.07	−0.12	−0.04	−0.01	−0.15*	0.13	0.04	0.09	0.84	0.06	0.11	−0.08	0.02
Concern*_POS_*	0.15*	0.16*	0.13	−0.06	0.009	−0.08	−0.05	0.09	0.05	0.89	0.34	0.50	0.20
Concern*_NEG_*	0.13	0.002	−0.004	−0.03	0.10	0.35***	0.23***	0.18**	0.09	0.30***	0.87	0.37	0.38
Arousal*_POS_*	0.23***	0.16*	0.11	−0.04	0.03	−0.07	−0.04	−0.02	−0.07	0.45***	0.33***	0.91	0.27
Arousal*_NEG_*	0.08	−0.04	0.03	−0.02	0.14*	0.25***	0.21**	0.02	0.02	0.18**	0.34***	0.25***	0.93

To assist comparing correlation estimates, the upper triangle of the matrix presents the disattenuated correlations (Spearman, 1904). Note that the confidence intervals around these estimates are also corrected for attenuation, so while the point estimate increases so does the width of the confidence interval (also meaning no change in statistical significance). The diagonal presents the reliability estimates. For single-item measures, where reliability cannot be estimated, we input ‘NA’. *p < 0.05, **p < 0.01, ***p < 0.001. IAT, Race-based Implicit Association Test; Warmth_W_, explicit White warmth; Preference_W_, explicit White preference; Imitation, imitative tendencies; eRec, emotion recognition; EC, self-reported empathic concern; PT, perspective taking; PD, personal distress; VPT, level-1 visual perspective taking; Concern_POS_ and Concern_NEG_, empathic concern toward positive and negative valence images; Arousal_POS,_ Arousal_NEG_, arousal toward positive and negative valence images.

### Discussion

Experiment 1 examined whether individual differences in race-IAT scores are related to other measures of implicit and explicit social cognition. In line with previous findings (Nosek et al., 2002; Nosek B. et al., 2007; Lai et al., 2014), the sample expressed primarily pro-White/anti-Black implicit attitudes, and this varied systematically both between and within participant race. Specifically, while the majority of White and Asian participants were classified as expressing weak or moderate pro-White/anti-Black bias, a large proportion of Black participants were classified as expressing weak pro-Black or weak pro-White bias. This is consistent with previous research suggesting that racial identity moderates IAT performance and may suggest that this task has construct validity as a measure of individual differences in racial attitudes (Rae and Greenwald, 2017).

Findings also revealed that participants exhibiting a moderate pro-White/anti-Black implicit bias reported greater explicit positive arousal toward White task actors relative to individuals showing weak pro-White and weak pro-Black implicit biases. This may be indicative of a positive in-group bias wherein those expressing pro-White implicit bias also express positive affective empathy toward this same racial group (Brown et al., 2006; Stürmer et al., 2006). However, differences between IAT clusters were statistically equivalent for implicit indices of imitative tendencies, emotion recognition, and visual perspective taking, as well as explicit measures of empathic concern and negative arousal. Differences in self-reported perspective taking, personal distress and positive and negative empathic concern were inconclusive. As such, although we found dissociable clusters of race-IAT scores, there were few differences among these clusters on the other measures of social cognition. The low reliability of the SRC task may have attenuated relationships between the race-IAT and imitative tendencies, but the same cannot be argued for the remaining tasks because these showed satisfactory reliability.

It could be argued that these findings are limited by the statistical power determined by the sample sizes of each cluster. This was overcome by performing sample-wide correlational analyses to further assess relationships between race-IAT scores and our measures of social cognition. This revealed that race-IAT scores were positively related to explicit positive arousal for White task actors, predicting additional variance from indices of explicit racial bias. Except the relationship between the race-IAT and negative concern which was inconclusive, relationships between race-IAT scores and other indices of social cognition were statistically equivalent. Taken together, these findings suggest that individual differences in the race-IAT are related minimally to other measures of implicit social cognition as well as to explicit trait and negative state affective empathy. This is contrary to recent theoretical propositions which suggest that the race-IAT should be related more strongly to other implicit rather than explicit measures of social cognition (Brownstein et al., 2020).

We do, however, remain tentative about the findings from Experiment 1 because of an important limitation: the lack of conceptual correspondence between the race-IAT and our other measures of social cognition. Specifically, while the race-IAT provides a relative measure of performance by comparing responses to White and Black actors, the behavioral tasks employed to measure social cognition featured White actors exclusively. Although previous studies have found relationships between the race-IAT and absolute indices of social behavior (see Greenwald et al., 2009a), recent research suggests that relative criterion measures produce stronger relationships (Brownstein et al., 2019; Gawronski, 2019; Kurdi et al., 2019). Furthermore, this means that we cannot be certain that participants show similar racial biases on these measures of imitative tendencies, emotion recognition, VPT and affective empathy as they do on the race-IAT. From Experiment 1, a question remained – does the IAT lack construct validity as a measure of individual differences in implicit social cognition or are the other measures we employed insensitive to racial bias? Experiment 2 mitigated this limitation.

## Experiment 2

### Materials and methods

#### Data availability and transparency statement

The experimental materials, code, and anonymized data are publicly available on the Open Science Framework.^3^ This study was preregistered *via* the standard Open Science Framework template prior to data collection and analysis.^4^ Deviations to this preregistration are outlined explicitly herein and do not change the interpretation of results. Additional methodological details are provided in the Supplementary material.

#### Participants

The sample size was determined using the same rationale as Experiment 1. A total of 257 participants were recruited, but 15 were excluded due to missing data, 4 owing to poor performance, and 1 who reported their race as ‘other’. The final sample comprised 237 participants (179 females; *M*_AGE_ = 22.00, *SD* = 7.36) of whom 184 identified as White, 22 Black, and 31 Asian. Sensitivity power analyses indicated that this sample had 80% power to detect two-tailed correlations of *r* > 0.18.

#### Measures and procedure

To ensure the other measures of social cognition had conceptual correspondence with the race-IAT, we adapted each of the tasks outlined in Experiment 1 to feature White and Black actors (refer to Figure 1). The IRI was included as an exploratory self-report measure. Dependent variables for indices of imitative tendencies, emotion recognition, VPT and affective empathy were calculated in the same way as Experiment 1 but for White and Black task actors separately. Reliability was within an acceptable range for emotion recognition (split-half White actor = 0.78, Black = 0.71), VPT (White = 0.72, Black = 0.78), Concern*_POS_* (White = 0.77, Black = 0.89), Concern*_NEG_* (White = 0.72, Black = 0.80), Arousal*_POS_* (White = 0.92, Black = 0.88), and Arousal*_NEG_* (White = 0.89, Black = 0.93), as well as for self-reported PT (*Ω* = 0.77), EC (*Ω* = 0.76) and PD (*Ω* = 0.78). However, it was again low for imitative tendencies (White = 0.12, Black = 0.15). To allow for correlations with the race-IAT, a mean difference score was then calculated from each of these task variables, with positive scores representing pro-White/anti-Black bias and negative scores representing pro-Black/anti-White bias. Scores in either of these directions therefore indicate *greater* socio-cognitive processing for that task actor. For these dependent variables, reliability was in a satisfactory range for the race-IAT (splithalf = 0.95) but was low for imitative tendencies (splithalf −0.03), emotion recognition (splithalf = 0.11), VPT (splithalf = 0.0), Concern*_POS_* (splithalf = 0.25), Concern*_NEG_* (splithalf = −0.40), Arousal*_POS_* (splithalf = 0.12), and Arousal*_NEG_* (splithalf = −0.30). These reliability analyses were suggested by two previous peer reviewers and performed after we had conducted our preregistered analyses. We therefore report the full results of our analyses herein, include correlations both corrected and uncorrected for this task measurement error, and discuss the limitations that arise from low task reliability in detail within the General Discussion.

#### Analytic strategy

Participants achieving 50% accuracy or less on accuracy-based tasks (SRC task, *n* = 23; Dot task, *n* = 9) and those with too many missing conditions/trials (SRC task, *n* = 13, Dot task, *n* = 9) were excluded from analyses. Performance checks indicated that each task performed as expected; IAT scores differed significantly from zero and showed a pro-White/anti-Black bias (*M* = 0.29, *SD* = 0.35, *p* < 0.001) and this was positively related to explicit Warmth*_W_*. RTs were greater on Incompatible relative to Compatible trials of the SRC task (*p* < 0.001), and on Inconsistent compared with Consistent trials of the Dot task (*p* < 0.001); and affective empathy was greater for negative relative to positive valence images on the SAE (*p* < 0.001). In our preregistration, we first planned to assess whether our selected measures of social cognition were able to detect racial biases by conducting a series of 3 (Participant Race: White, Black, Asian [B-S]) x 2 (Actor Race: White, Black [W-S]) mixed-design ANOVAs on imitative tendencies, emotion recognition, and visual perspective taking, with an additional two-level within-factor of Valence (Positive, Negative [W-S]) for empathic concern and arousal. However, despite advertising this study to the Black, Asian, and Minority Ethnic (BAME) student societies, the final sample size of Black (*n* = 20) and Asian participants was small (*n* = 29), meaning that such analyses would only be able to reliably detect moderate-to-large differences (*d* > 0.59) with 80% power. These analyses are reported in the Supplementary material and do not change the interpretation of the results reported herein. As a deviation to these preregistered analyses, we instead report exploratory paired samples *t*-tests for White and Black participants only by collapsing across participant race and instead assessing whether these participants expressed an own- or other-race bias for the White and Black actors depicted in the social cognition tasks. This provided 80% statistical power to detect small differences using two-tailed paired-samples *t*-tests (lowest *n* = 185; *d_z_ > 0.*21). As planned, we then report correlations between performance on the race-IAT and our other measures of social cognition.

### Results

Participants exhibited better emotion recognition toward own-race (*M* = 0.93, *SD* = 0.07) relative to other-race actors (*M* = 0.92, *SD* = 0.08, *p* = 0.014, *d_z_* = 0.18), and greater Concern*_POS_* toward own- (*M* = 11.73, *SD* = 6.89) relative to other-race actors (*M* = 10.29, *SD* = 6.33, *p* < 0.001, *d_z_* = 0.36). However, they exhibited quicker perspective taking toward other-race (*M* = 837.62, *SD* = 183.03) relative to own-race actors (*M* = 878.80, *SD* = 13.17, *p* < 0.001, *d_z_* = 0.31) as well as higher Concern*_NEG_* (*M^-OTHER^* = 48.97, *SD* = 11.88 vs. *M^-OWN^* = 47.05, *SD* = 11.38, *p* < 0.001, *d_z_* = 0.34) and Arousal*_NEG_* (*M^-OTHER^* = 29.74, *SD* = 17.10 vs. *M^-OWN^* = 28.21, *SD* = 16.16, *p* < 0.001, *d_z_* = 0.26). Differences in imitative tendencies (*p* = 0.56, *d_z_* = −0.04) and Arousal*_POS_* (*p* = 0.93, *d_z_* = 0.0) between own- and other-race actors were statistically equivalent. This provided an indication that our indices of emotion recognition, perspective taking, positive and negative empathic concern and positive arousal were sensitive in detecting racial biases, however indices of imitative tendencies and positive arousal were not.

Correlational analyses indicated that both race-IAT scores and Warmth*_W_* showed a positive relationship with relative ratings of Arousal*_POS_*, suggesting that higher pro-White/anti-Black bias was related to higher explicit positive arousal for White relative to Black task actors. Follow-up regression analyses indicated that race-IAT scores and Warmth*_W_* together explained 11.9% of the variance in Arousal*_POS_*, *F*(1, 235) = 17.52, *p* < 0.001, with IAT scores explaining an additional 5% of variance, *p* < 0.001. Relationships between the race-IAT and implicit measures of imitative tendencies, emotion recognition, and visual perspective taking, as well as explicit state and trait affective empathy were statistically equivalent. Table 3 presents the correlation matrix.

**Table 3:** Correlation matrix showing relationships between implicit and explicit racial bias and social cognition in Experiment 2.

	IAT	Warmth*_W_*	Preference*_W_*	Imitation	eRec	VPT	Concern *POS*	Concern *NEG*	Arousal *POS*	Arousal NEG	EC	PT	PD
IAT	0.95	0.16	0.07	-	−0.09	-	0.12	-	0.28	-	0.04	0.04	−0.12
Warmth*_W_*	0.16*	NA	0.62	-	0.18	-	0.42	-	0.28	-	−0.07	−0.11	0.00
Preference*_W_*	0.07	0.62***	NA	-	−0.02	-	0.10	-	0.12	-	−0.10	−0.10	−0.09
Imitation	−0.04	0.08	0.10	−0.03	-	-	-	-	-	-	-	-	-
eRec	−0.03	0.06	−0.006	−0.006	0.11	-	0.48	-	0.13	-	−0.28	0.03	−0.01
VPT	−0.03	0.03	0.09	−0.06	−0.004	0	-	-	-	-	-	-	-
Concern*_POS_*	0.06	0.21**	0.05	0.16*	0.08	−0.04	0.25	-	0.61	-	0.02	0.21	0.20
Concern*_NEG_*	0.003	0.01	−0.009	−0.06	0.05	0.10	0.09	−0.40	-	-	-	-	-
Arousal*_POS_*	0.26***	0.26***	0.11	0.02	0.04	0.01	0.29***	0.09	0.12	-	−0.16	−0.08	−0.04
Arousal_NEG_	0.03	0.05	−0.03	−0.05	0.09	−0.06	−0.07	0.20**	0.06	−0.03	-	-	-
EC	0.03	−0.06	−0.09	−0.07	−0.08.	0.17	0.01	0.05	−0.13*	0.007	0.76	0.63	0.29
PT	0.03	−0.10	−0.09	−0.05	0.01	0.11	0.09	0.05	−0.07	−0.10	0.48***	0.77	−0.12
PD	−0.10	0.004	−0.08	−0.03	−0.002	0.03	0.09	0.07	−0.03	0.009	0.22**	−0.09	0.78

To assist comparing correlation estimates, the upper triangle of the matrix presents the disattenuated correlations (Spearman, 1904). Note that the confidence intervals around these estimates are also corrected for attenuation, so while the point estimate increases so does the width of the confidence interval (also meaning no change in statistical significance). The diagonal presents the reliability estimates. We omit disattenuated estimates using measures with reliability estimates equal to or below zero as these result in non-sensical correlation estimates often exceeding 1 and reaching infinity. For single-item measures, where reliability cannot be estimated, we input ‘NA’. *p < 0.05, **p < 0.01, ***p < 0.001. All variables are expressed as relative indices with positive scores indicating biases in socio-cognitive processing toward a White relative to Black actor. The IRI sub-scales of EC, PT, and PD were included as exploratory measures.

### General discussion

Guided by three main theoretical debates in the IAT literature to date, the current study aimed to assess whether individual differences in race-IAT performance are related to other implicit (and explicit) socio-cognitive processes that are proposed guide social interaction; namely, imitative tendencies, emotion recognition, perspective taking and affective empathy. On the basis of prior research which suggests that the IAT is an implicit measure of social cognition, we expected participants expressing stronger pro-White/anti-Black implicit bias to show enhanced socio-cognitive processing of White task actors in Experiment 1 and White relative to Black task actors in Experiment 2. Across both of these experiments, we found that greater expressions of pro-White/anti-Black bias were associated with greater explicit positive arousal for White task actors; a measure of state affective empathy. However, race-IAT performance was not associated reliably with differences in implicit measures of imitative tendencies, emotion recognition, and visual perspective taking, as well as explicit trait and negative state affective empathy. We now discuss these findings centring on the three main questions surrounding the use of the race-IAT, before exploring possible alternative explanations and how these can guide future research.

To what extent does the IAT measure racial biases that are *implicit* in nature? One theoretical premise of the IAT is that it is capable of assessing the automatic activation of attitudes that are either easily concealed or inaccessible to conscious introspection (Greenwald et al., 1998; Lane et al., 2007; Nosek et al., 2011; Greenwald and Lai, 2020). Findings from Experiment 1 revealed that individual differences in implicit racial bias are positively correlated with explicit racial bias, but cluster analyses reveal a more nuanced picture. Specifically, participants expressing weak pro-Black/anti-White bias (C#1) reported less warmth for White people and a slight preference for Black people compared to those expressing weak pro-White/anti-Black bias (C#2) who showed relative neutrality on these measures. This pattern of findings suggests that weak expressions of implicit racial bias correspond to explicit self-reports. However, participants who expressed moderate pro-White/anti-Black bias (C#3) on the race-IAT also reported relatively neutral explicit racial bias which did not significantly differ to those showing weak pro-White/anti-Black bias. This may therefore suggest that some people are likely to conceal, or be unable to introspect on, their racial attitudes, with the race-IAT therefore providing a more sensitive measure of individual differences in racial bias. Nevertheless, performance on the race-IAT was only consistently related to measures of explicit positive affective empathy and was not related to implicit (indirect) measures of imitative tendencies, emotion recognition, and perspective taking. This is contrary to previous research which theoretically predicts that the race-IAT should be related more strongly to other implicit relative to explicit measures of social cognition (Brownstein et al., 2020). Other studies have also begun to question whether there is anything implicit about the IAT; research consistently shows that participants show awareness of, and control over, their performance on this measure (Vanman et al., 2004; Röhner et al., 2013; Hahn et al., 2014), and the index of racial bias emerging from the race-IAT appears to map onto the same latent construct as explicit measures (Schimmack, 2021; Vianello and Bar-Anan, 2021). Our findings converge with the results of these studies, revealing relationships between the race-IAT and explicit – but not implicit – measures of social cognition. This is perhaps unsurprising given that participants are made *explicitly* aware of the target constructs (White, Black, Good, Bad) that appear throughout race-IAT trials. It might be the case, therefore, that explicit categorization processes contribute to race-IAT effects (see also Yamaguchi and Beattie, 2020).

Does the IAT measure individual differences as a facet of *social cognition*? The cognitive mechanisms involved in processing socially relevant information are subsumed under the umbrella construct of social cognition and are believed to operate largely beyond conscious awareness (Frith and Frith, 2012; Happé et al., 2017). We therefore rationalized that should implicit biases reflect the socio-cognitive processes at the heart of social perception, judgment and action (Greenwald et al., 1998; Kurdi and Banaji, 2017; Rae and Greenwald, 2017), then performance on the IAT should be related to other socio-cognitive processes that guide how we interact with others (see also Nosek et al., 2011). Across both experiments, we found that race-IAT performance was reliably related to positive arousal for White task actors, explaining incremental predictive validity above explicit self-reports. Greater expressions of pro-White/anti-Black bias may thus be related to feelings of positive arousal (i.e., excitement) for White people, a facet of affective empathy that allows us to share in other’s positive emotional states (Spence and Townsend, 2008; Shamay-Tsoory and Lamm, 2018). However, we found that relationships between race-IAT performance and imitative tendencies, emotion recognition, visual perspective taking, and both trait and state negative affective empathy were statistically equivalent. The question therefore remains if the race-IAT represents a measure of individual differences in social cognition; is it unclear why performance on this task was not related to other socio-cognitive processes that are believed to operate largely beyond conscious awareness.

Another remaining question in the current literature to date concerns whether individual differences in implicit racial bias can predict social behavior. Previous meta-analyses have revealed weak correlations between race-IAT performance and explicit measures of intergroup discrimination (Greenwald et al., 2009a; Oswald et al., 2013; Kurdi and Banaji, 2017; Kurdi et al., 2019), which may be explained by the latter being equally susceptible to self-presentational motives and social desirability as explicit self-reports (Brownstein et al., 2020). Researchers have therefore argued that it is unlikely that implicit measures, such as the race-IAT will correlate strongly with behavioral outcomes measured explicitly (Brownstein et al., 2020) and have suggested that individual differences on this task should be related to other implicit measures of social cognition that are less accessible or amenable to conscious control (Nosek et al., 2011; Gawronski, 2019). Nevertheless, in the current study we found that the race-IAT was not related to other indices of social behavior that were measured implicitly. Taken together then, the results of the current study may pose further questions for the construct and predictive validity of the race-IAT: is it a measure of individual differences in social cognition and to what extent can performance on this task predict social behavior?

There are, however, alternative possible explanations for these findings. First, we attempted to validate the race-IAT by comparing performance on this task with other measures of social cognition used commonly, but this assumes that these latter tasks are also capable of detecting racial biases. In Experiment 1 we used absolute measures of imitative tendencies, emotion recognition, perspective taking and state affective empathy with participants responding to only White task actors. This meant, however, that our tasks lacked conceptual correspondence with the race-IAT, which is a relative measure of implicit racial biases between White *and* Black task actors. We overcame this limitation in Experiment 2 by adapting the tasks comprising our assessment battery to feature both White and Black actors. Findings indicated that participants exhibited better emotion recognition and greater positive concern toward own- relative to other-race actors, and showed quicker perspective taking, negative concern and negative arousal to other- relative to own-race actors. This might reveal cognitive mechanisms through which social categorization can exert influences over social behavior. Furthermore, this indicated that these measures of social cognition were indeed capable of detecting racial biases and could theoretically be related to performance on the race-IAT. However, differences in imitative tendencies and positive arousal were not modulated by racial group membership.

Estimates of split-half reliability were also low for imitative tendencies in both experiments and for the difference scores for emotion recognition, visual perspective taking, and affective empathy in Experiment 2. Estimating split-half reliability is imperative for individual differences research and low reliability attenuates the strength of relationships between variables (Parsons et al., 2019). This may explain why we found minimal relationships between the race-IAT and other measures of social cognition, suggesting that our selection of measures was not entirely suitable for capturing individual differences. Similarly, because trials were split between White and Black task actors in Experiment 2, each dependent variable comprised a lower number of experimental trials, which could have further affected task reliability. A wealth of research employs these tasks to measure individual differences in social cognition and task reliability is rarely reported. If other work corroborates the very low reliability we report here then we will likely, as a field, need to invest in developing measures more psychometrically suited to addressing questions surrounding individual differences.

Finally, the lack of relationships between the race-IAT and the measures of social cognition that we have employed may be explained by each of these constituent tasks relying on, to a greater or lesser extent, other (non-social) cognitive or attentional processes. Recently it has been shown that the Stimulus Response Compatibility task, which is used commonly to measure imitative tendencies (Cracco et al., 2018), captures more general-purpose cognitive processes deployed in both social *and* non-social contexts, such as response inhibition and interference resolution (Czekóová et al., 2021; Rauchbauer et al., 2021). Furthermore, Santiesteban et al. (2012) provide evidence that the Dot Task relies on the directional rather than agentive features of the task avatar, suggesting that performance on this task may reflect automatic attentional orienting rather than perspective taking *per se*. Interestingly, similar findings have been demonstrated for the race-IAT; non-associative, non-attitudinal processes such as inhibition and shifting appear to influence performance on this task (see Calanchini et al., 2014). Together, this aligns with an emerging literature that suggests many socio-cognitive processes may actually reflect non-social, domain-general mechanisms (Darda et al., 2020; Shaw et al., 2020).

With these limitations in mind, it may be reasonably argued that the current study is unable to answer the initial research questions regarding the race-IAT. Nevertheless, given that this cannot be achieved by a single study and that publication bias is substantial within the psychological literature (see Scheel et al., 2021), we hope that researchers can learn from these findings and that they inform future work which sets out to answer these and similar questions.

## Conclusion

The IAT was coined as an individual difference measure of *implicit social cognition* (Greenwald et al., 1998; Nosek et al., 2011), yet empirical evidence that supports its relationships with other measures of implicit social cognition is lacking. This study assessed whether individual differences in race-IAT performance are related to other implicit and explicit socio-cognitive processes that are proposed to guide social interaction. Across two experiments, findings indicate that implicit racial bias was associated reliably with explicit positive arousal for White task actors (a facet of state affective empathy) and explained incremental predictive validity above explicit self-reports. However, there were no significant relationships between the race-IAT and implicit imitative tendencies, emotion recognition, visual perspective taking, and explicit trait and negative state affective empathy. These findings are in contrast to recent propositions, which propose that the race-IAT should correlate more strongly with other implicit relative to explicit processes that are subsumed within social cognition. Future research is therefore required to explore empirical support for the theoretical tenets of the race-IAT, which is an important endeavor given the influence that this measure has had within policy, politics, and public discourse.

## Data availability statement

The datasets presented in this study can be found in online repositories. The names of the repository/repositories and accession number(s) can be found below: All materials, data and supporting information are publicly available for Experiment 1 (https://osf.io/a469p/) and Experiment 2 (https://osf.io/m46jn/). Experiment 2 was preregistered *via* the standard Open Science Framework template (https://osf.io/bmjz6) and any deviations are explicitly stated within this manuscript.

## Ethics statement

Written informed consent was obtained from the individuals for the publication of any potentially identifiable images included in this article.

## Author contributions

CP: conceptualization, methodology, data curation, formal analysis, funding acquisition, project administration, resources, supervision, writing—original draft, and review and editing. MP, PM, NN, and CB: investigation, project administration, resources, software, and writing—review and editing. SP: validation, formal analysis, and writing—review and editing. DS: conceptualization, methodology, data curation, project administration, resources, software, supervision, validation, visualization, and writing—review and editing. All authors contributed to the article and approved the submitted version.

## Funding

This research was supported by an internal Early Career Researcher Award received by CP.

## Acknowledgments

The authors would like to thank two previous reviewers who provided substantial and thoughtful comments and recommended that we should compute split-half reliability for the experimental tasks used in this research.

## Conflict of interest

The authors declare that the research was conducted in the absence of any commercial or financial relationships that could be construed as a potential conflict of interest.

## Publisher’s note

All claims expressed in this article are solely those of the authors and do not necessarily represent those of their affiliated organizations, or those of the publisher, the editors and the reviewers. Any product that may be evaluated in this article, or claim that may be made by its manufacturer, is not guaranteed or endorsed by the publisher.

## Footnotes

^1^ https://osf.io/a469p/

^2^It was due to a computer error that participants only saw 13 negative images.

^3^ https://osf.io/m46jn/

^4^ https://osf.io/bmjz6
