# Supplementary material for: Relationships between the race implicit association test and other measures of implicit and explicit social cognition
Source: Front Psychol. 2023 Jul 27;14:1197298. doi: 10.3389/fpsyg.2023.1197298 (PMC10415041; doi:10.3389/fpsyg.2023.1197298)
Supplement: Supplementary file 1 [file Table_1.DOCX]

Supplementary Material

**Is the race Implicit Association Test related to other individual difference measures of social cognition?**

Charlotte R. Pennington^1*^, Matthew Ploszajski^2^, Parmesh Mistry^1^, Nicola NgOmbe^1^, Charlotte Back^2^ , & Daniel J. Shaw^1^

*Correspondence: Dr Charlotte R. Pennington, [c.pennington@aston.ac.uk](mailto:c.pennington@aston.ac.uk)

The information below provides further detailed information regarding the methodology and analysis results associated with this manuscript.

**Methods**

For both experiments, volunteers were recruited to take part in a study ostensibly investigating relationships between different measures of social cognition. In a multi-station lab separated by dividers, participants were tested in groups of four or less and seated approximately 57cm from the computer monitor. On-screen instructions were presented before each task and breaks were provided throughout.

**Experiment 1**

**Imitative Tendencies**

Participants completed two blocks of the stimulus response compatibility task (SRC), but due to a technical error, the blocks were not counterbalanced: Since a high number of participants achieved less than 50% accuracy on the first block depicting a right stimulus hand, we only report responses on the second block of 72 trials depicting a left hand, for which accuracy was high (*M* = 90%) and RTs demonstrated acceptable reliability.

Each trial began with the stimulus hand resting on a flat surface, signalling that participants should depress both the left and right directional arrows on the computer keyboard with their index and middle finger of their right hand, respectively. After a stimulus onset asynchrony of 800, 1600, or 2400ms, selected randomly, the stimulus hand then changed to the endpoint of either an index- or middle-finger extension, and a coloured dot was presented between these two fingers. The colour of the dot signalled whether the participant should extend their own index (green) or middle (red) finger. In response to the imperative stimulus, participants lifted the required finger as quickly as possible, thereby releasing the corresponding key. A blank screen was then presented for 1000ms between trials.

**Experiment 2**

For each task, dependent variables were calculated for responses to White and Black task actors separately and then a mean difference was calculated with positive scores representing pro-White/anti-Black bias and negative scores representing pro-Black/anti-White bias.

**Imitative Tendencies**

The SRC task comprised two blocks of 72 randomised trials. Each block presented either a White or Black actor’s hand and block order was counterbalanced across participants. To overcome potential orthogonal compatibility effects in Experiment 1 (see Shaw et al., 2017), we used only a right stimulus hand rotated 90° clockwise from the participants’ perspective (*a* > .70). A measure of automatic imitation in response to the White and Black actor was computed separately by subtracting mean RT on Compatible from Incompatible trials within each block, with higher scores representing greater imitative tendencies.

**Emotion Recognition**

The eGNG task comprised White and Black actor’s faces selected from the NimStim database, split evenly among both actor race and gender. The task comprised six blocks of 40 trials, with the same three emotional ‘Go’ blocks and three non-emotional ‘Go’ blocks as Experiment 1. The same measure of hit rate was calculated, but separately for White and Black faces. Higher scores represent better emotion recognition.

**Visual Perspective Taking**

The Dot task was adapted to feature a White or Black female actor. In Experiment 1, the gender of the actor was matched to that of the participant, but in Experiment 2, gender was kept constant so that only racial group membership was manipulated^[[1]](#footnote-1)^. The task comprised four blocks of 52 randomised trials, as in Experiment 1, but the first two blocks always featured a White actor while the final two blocks featured a Black actor (*a* > .68). The single-dimension index was calculated for the White and Black actor separately by averaging RT on Other-Inconsistent match trials, with higher values indicating poorer perspective taking ability.

**State Affective Empathy**

The SAE task was adapted to measure empathic concern and arousal towards both White and Black actors. We selected 38 photographs from the IAPS depicting an equal split of White and Black people expressing negative (*n* = 14), positive (*n* = 14) and neutral (*n* = 10) emotions in various contexts. Importantly, normative IAPS ratings were used to ensure that valence and arousal differed significantly between positive and negative images (all *p* < .001) but did not differ significantly as a function of actor race (all *p* > .05). In line with Experiment 1, total scores for empathic concern and arousal (*a* > .90) were derived for ratings of positive and negative images of White and Black actors separately (Concern*_POS_*, Concern*_NEG_*, Arousal*_POS_*, and Arousal*_NEG_*).

**Supplementary Statistical Analyses**

**Experiment 1: Task Performance Checks**

*Implicit Association Test (IAT)*

A one-sample *t*-test indicated that IAT scores significantly differed from zero, with participants exhibiting a weak Pro-White/Anti-Black bias (IAT *d, M* = .32, *SD* = .36), *t*(225) = 13.48, *p* < .001, Cohen’s *d* = .89.

*Imitative Tendencies*

A paired-samples *t*-test indicated that RTs were quicker on Compatible (*M* = 560.32, *SD* = 73.90) relative to Incompatible trials on the SRC task (*M* = 584.02, *SD* = 79.18), *t*(212) = -8.25, *p* < .001, *d_z_* = .56.

*Visual Perspective Taking*

A 2 (Perspective: Self vs. Other) x 2 (Consistency: Consistent vs. Inconsistent) repeated-measures Analysis of Variance (ANOVA) indicated a main effect of Perspective, *F*(1, 211) = 38.79, *p* < .001, *η_p_^2^* = .16, and Consistency, *F*(1, 211) = 198.47, *p* < .001, *η_p_^2^* = .49, on the Dot task. Participants were quicker to respond accurately on Self (*M* = 786.57, *SD*^[[2]](#footnote-2)^ = 148.08) relative to Other trials (*M* = 819.61, *SD* = 161.33), *p* < .001, *d_z_* = .43, and on Consistent (*M* = 761.04, *SD* = 148.22) compared with Inconsistent trials (*M* = 845.15, *SD* = 163.66), *p* < .001, *d_z_* = .97.

*Affective Empathy*

*Empathic Concern.* A paired-samples *t-*test indicated that empathic concern was greater for negative (*M* = 74.12, *SD* = 19.17) relative to positive images (*M* = 23.65, *SD* = 11.99), *t*(225) = 39.33, *p* < .001, *d_z_* = 2.61.

*Arousal.* A paired-samples *t-*test indicated that arousal was greater for negative (*M* = 47.48, *SD* = 24.16) relative to positive images (*M* = 38.23, *SD* = 20.10), *t*(225) = 5.08. *p* < .001, *d_z_* = .34.

**Experiment 1: Equivalence Test Outputs**

***Cluster Analyses***

***Explicit Racial Bias***

*White Warmth*. The observed effect size (*p* = .07, *d* = .35) between C#2 (*M* = -.31, *SD* = 1.75) and C#3 (*M* = .17, *SD* = .94) was inconclusive.

| **The TOST procedure based on Welch's t-test indicated that the observed effect size (d = -0.35) was not significantly within the equivalent bounds of d = -0.5 and d = 0.5, (or in raw scores: -0.7 and 0.7), t(119.47) = 1.05, p = 0.147** |
| --- |
|  |
|  |

*White Preference.* The observed effect size (*p* = .07, *d* = .43) between C#2 (*M* = 1.95, *SD* = .34) and C#3 (*M* = 2.07, *SD* = .35) was inconclusive.

| **The TOST procedure based on Welch's t-test indicated that the observed effect size (d = -0.43) was not significantly within the equivalent bounds of d = -0.5 and d = 0.5, (or in raw scores: -0.17 and 0.17), t(181.1) = 0.45, p = 0.326** |
| --- |
|  |
|  |

***Implicit Racial Bias & Social Cognition***

***Positive arousal***

The observed effect size (*p* = .94, *d* = .42) between C#1 (*M* = 2.19, *SD* = 1.11) and C#2 (*M* = 2.48, *SD* = 1.19) was inconclusive.

| **The TOST procedure based on Welch's t-test indicated that the observed effect size (d = -0.42) was not significantly within the equivalent bounds of d = -0.5 and d = 0.5, (or in raw scores: -0.7 and 0.7), t(190.78) = 0.47, p = 0.318** |
| --- |
|  |
|  |

***Imitative Tendencies***

There was no significant difference in imitative tendencies between C#1 (*M* = 25.05, *SD* = 47.81) and C#2 (*M* = 27.14, *SD* = 42.77), *p* > .05, with the observed effect size (*d* = -.05) statistically within the equivalence bounds. There was also no significant difference between C#2 and C#3 (*M* = 20.66, *SD* = 39.70), with the observed effect size (*d* = .16) significantly within the equivalence bounds. There was no significant difference between C#1 and C#3, with the observed effect size (*d* = .11) significantly within the equivalence bounds.

| **The TOST procedure based on Welch's t-test indicated that the observed effect size (d = -0.05) was significantly within the equivalent bounds of d = -0.5 and d = 0.5, (or in raw scores: -22.68 and 22.68), t(42.9) = 2.02, p = 0.025** |
| --- |
|  |
|  |
| **The TOST procedure based on Welch's t-test indicated that the observed effect size (d = 0.16) was significantly within the equivalent bounds of d = -0.5 and d = 0.5, (or in raw scores: -20.63 and 20.63), t(165.51) = -2.3, p = 0.011** |
|  |
|  |
| **The TOST procedure based on Welch's t-test indicated that the observed effect size (d = 0.11) was significantly within the equivalent bounds of d = -0.5 and d = 0.5, (or in raw scores: -21.97 and 21.97), t(37.62) = -1.79, p = 0.041** |
|  |
|  |

***Emotion Recognition***

There was no significant difference in emotion recognition between C#1 (*M* = .88, *SD* = .08) and C#2 (*M* = .87, *SD* = .07), *p* > .05, with the observed effect size (*d* = .14) statistically within the equivalence bounds. There was also no significant difference between C#2 and C#3 (*M* = .87, *SD* = .06), with the observed effect size (*d* = .0) significantly within the equivalence bounds. There was no significant difference between C#1 and C#3, with the observed effect size (*d* = .15) significantly within the equivalence bounds.

| **The TOST procedure based on Welch's t-test indicated that the observed effect size (d = 0.14) was significantly within the equivalent bounds of d = -0.5 and d = 0.5, (or in raw scores: -0.04 and 0.04), t(52.32) = -1.74, p = 0.044** |
| --- |
|  |
|  |
| **The TOST procedure based on Welch's t-test indicated that the observed effect size (d = 0) was significantly within the equivalent bounds of d = -0.5 and d = 0.5, (or in raw scores: -0.03 and 0.03), t(163.35) = -3.41, p = 0** |
|  |
|  |
| **The TOST procedure based on Welch's t-test indicated that the observed effect size (d = 0.15) was significantly within the equivalent bounds of d = -0.5 and d = 0.5, (or in raw scores: -0.04 and 0.04), t(43.45) = -1.68, p = 0.05** |
|  |
|  |

**Trait Empathy**

***Self-reported Empathic Concern (EC)***

There was no significant difference in self-reported empathic concern between C#1 (*M* = 21.42, *SD* = 4.44) and C#2 (*M* = 20.98, *SD* = 4.87), *p* > .05, with the observed effect size (*d* = .09) statistically within the equivalence bounds. There also was no significant difference between C#2 and C#3 (*M* = 20.92, *SD* = 4.41), with the observed effect size (*d* = .01) significantly within the equivalence bounds. There was no significant difference between C#1 and C#3, with the observed effect size (*d* = .11) significantly within the equivalence bounds.

| **The TOST procedure based on Welch's t-test indicated that the observed effect size (d = 0.09) was significantly within the equivalent bounds of d = -0.5 and d = 0.5, (or in raw scores: -2.33 and 2.33), t(63.89) = -2.02, p = 0.024** | |
| --- | --- |
|  |  |
|  |  |
| **The TOST procedure based on Welch's t-test indicated that the observed effect size (d = 0.01) was significantly within the equivalent bounds of d = -0.5 and d = 0.5, (or in raw scores: -2.32 and 2.32), t(169.14) = -3.33, p = 0.001** | |
|  |  |
|  |  |
| **The TOST procedure based on Welch's t-test indicated that the observed effect size (d = 0.11) was significantly within the equivalent bounds of d = -0.5 and d = 0.5, (or in raw scores: -2.21 and 2.21), t(52.58) = -1.94, p = 0.029** |  |
|  |  |
|  |  |

***Self-reported Perspective Taking (PT)***

There was no significant difference in self-reported perspective taking between C#1 (*M* = 18.39, *SD* = 5.31) and C#2 (*M* = 18.21, *SD* = 4.46), *p* > .05, with the observed effect size (*d* = .04) statistically within the equivalence bounds. There also was no significant difference between C#2 and C#3 (*M* = 17.86, *SD* = 4.75), with the observed effect size (*d* = .08) significantly within the equivalence bounds. However, the non-significant difference between C#1 and C#3 was inconclusive (*d* = .31).

| **The TOST procedure based on Welch's t-test indicated that the observed effect size (d = 0.04) was significantly within the equivalent bounds of d = -0.5 and d = 0.5, (or in raw scores: -2.45 and 2.45), t(50.69) = -2.17, p = 0.017** |  |
| --- | --- |
|  |  |
|  |  |
| **The TOST procedure based on Welch's t-test indicated that the observed effect size (d = 0.08) was significantly within the equivalent bounds of d = -0.5 and d = 0.5, (or in raw scores: -2.3 and 2.3), t(183.67) = -2.93, p = 0.002** | |
|  |  |
|  |  |
| **The TOST procedure based on Welch's t-test indicated that the observed effect size (d = 0.31) was not significantly within the equivalent bounds of d = -0.5 and d = 0.5, (or in raw scores: -2.52 and 2.52), t(167.87) = -1.34, p = 0.091** | |
|  |  |
|  |  |

***Self-reported Personal Distress (PD)***

There was no significant difference in self-reported personal distress between C#1 (*M* = 13.42, *SD* = 4.12) and C#2 (*M* = 13.71, *SD* = 4.23), *p* > .05, with the observed effect size (*d* = -.07) statistically within the equivalence bounds. There also was no significant difference between C#2 and C#3 (*M* = 14.23, *SD* = 4.35), with the observed effect size (*d* = -.12) significantly within the equivalence bounds. However, the non-significant difference between C#1 and C#3 was inconclusive (*d* = -.19).

| **The TOST procedure based on Welch's t-test indicated that the observed effect size (d = -0.07) was significantly within the equivalent bounds of d = -0.5 and d = 0.5, (or in raw scores: -2.09 and 2.09), t(60.02) = 2.11, p = 0.02** |  |
| --- | --- |
|  |  |
|  |  |
| **The TOST procedure based on Welch's t-test indicated that the observed effect size (d = -0.12) was significantly within the equivalent bounds of d = -0.5 and d = 0.5, (or in raw scores: -2.15 and 2.15), t(181.02) = 2.61, p = 0.005** | |
|  |  |
|  |  |
| **The TOST procedure based on Welch's t-test indicated that the observed effect size (d = -0.19) was not significantly within the equivalent bounds of d = -0.5 and d = 0.5, (or in raw scores: -2.12 and 2.12), t(55.38) = 1.58, p = 0.06** | |
|  |  |
|  |  |

***Visual Perspective Taking***

There was no significant difference in visual perspective taking performance between C#1 (*M* = 863.93, *SD* = 153.36) and C#2 (*M* = 863.42, *SD* = 197.09), *p* > .05, with the observed effect size (*d* = .0) statistically within the equivalence bounds. There also was no significant difference between C#2 and C#3 (*M* = 886.18, *SD* = 183.88), with the observed effect size (*d* = -.12) significantly within the equivalence bounds. There was no significant difference between C#1 and C#3, with the observed effect size (*d* = -.13) significantly within the equivalence bounds.

| **The TOST procedure based on Welch's t-test indicated that the observed effect size (d = 0) was significantly within the equivalent bounds of d = -0.5 and d = 0.5, (or in raw scores: -88.29 and 88.29), t(72.99) = -2.51, p = 0.007** | |
| --- | --- |
|  |  |
|  |  |
| **The TOST procedure based on Welch's t-test indicated that the observed effect size (d = -0.12) was significantly within the equivalent bounds of d = -0.5 and d = 0.5, (or in raw scores: -95.3 and 95.3), t(163.91) = 2.53, p = 0.006** |  |
|  |  |
|  |  |
| **The TOST procedure based on Welch's t-test indicated that the observed effect size (d = -0.13) was significantly within the equivalent bounds of d = -0.5 and d = 0.5, (or in raw scores: -84.65 and 84.65), t(61.98) = 1.91, p = 0.031** |  |
|  |  |
|  |  |

**Affective Empathy**

***Positive concern***

There was no significant difference in positive concern between C#2 (*M* = 22.89, *SD* = 12.09) and C#3 (*M* = 25.13, *SD* = 12.39), *p* > .05, with the observed effect size (*d* = -.18) statistically within the equivalence bounds. However, the non-significant difference between C#1 (*M* = 20.73, *SD* = 9.84) and C#2 (*d* = -.19), and between C#1 and C#3 (*d* = -.37) was inconclusive.

| **The TOST procedure based on Welch's t-test indicated that the observed effect size (d = -0.18) was significantly within the equivalent bounds of d = -0.5 and d = 0.5, (or in raw scores: -6.12 and 6.12), t(180.74) = 2.19, p = 0.015** |
| --- |
|  |
|  |
| **The TOST procedure based on Welch's t-test indicated that the observed effect size (d = -0.19) was not significantly within the equivalent bounds of d = -0.5 and d = 0.5, (or in raw scores: -5.51 and 5.51), t(71.51) = 1.55, p = 0.063.** |
|  |
|  |
| **The TOST procedure based on Welch's t-test indicated that the observed effect size (d = -0.37) was not significantly within the equivalent bounds of d = -0.5 and d = 0.5, (or in raw scores: -5.59 and 5.59), t(65.61) = 0.57, p = 0.284.** |
|  |
|  |

***Negative Concern***

There was no significant difference between C#2 (*M* = 73.99, *SD* = 18.22) and C#3 (*M* = 75.62, *SD* = 19.25), with the observed effect size (*d* = -.09) significantly within the equivalence bounds. However, the non-significant difference between C#1 (*M* = 69.52, *SD* = 21.05) and C#2 (*d* = -.23) and C#1 and C#3 (*d* = -.31) was inconclusive.

| **The TOST procedure based on Welch's t-test indicated that the observed effect size (d = -0.09) was significantly within the equivalent bounds of d = -0.5 and d = 0.5, (or in raw scores: -9.37 and 9.37), t(183.09) = 2.86, p = 0.002** |
| --- |
|  |
|  |
| **The TOST procedure based on Welch's t-test indicated that the observed effect size (d = -0.23) was not significantly within the equivalent bounds of d = -0.5 and d = 0.5, (or in raw scores: -9.84 and 9.84), t(51.88) = 1.29, p = 0.102** |
|  |
|  |
| **The TOST procedure based on Welch's t-test indicated that the observed effect size (d = -0.31) was not significantly within the equivalent bounds of d = -0.5 and d = 0.5, (or in raw scores: -10.09 and 10.09), t(49.32) = 0.97, p = 0.168** |
|  |
|  |

***Negative Arousal***

There was no significant difference in negative arousal imitation between C#1 (*M* = 45.88, *SD* = 24.38) and C#2 (*M* = 45.54, *SD* = 22.35), *p* > .05, with the observed effect size (*d* = .01) statistically within the equivalence bounds. There was no significant difference between C#2 and C#3 (*M* = 49.47, *SD* = 25.46), with the observed effect size (*d* = -.16) significantly within the equivalence bounds. There was no significant difference between C#1 and C#3, with the observed effect size (*d* = -.14) significantly within the equivalence bounds.

| **The TOST procedure based on Welch's t-test indicated that the observed effect size (d = 0.01) was significantly within the equivalent bounds of d = -0.5 and d = 0.5, (or in raw scores: -11.69 and 11.69), t(54.33) = -2.32, p = 0.012** |
| --- |
|  |
|  |
| **The TOST procedure based on Welch's t-test indicated that the observed effect size (d = -0.16) was significantly within the equivalent bounds of d = -0.5 and d = 0.5, (or in raw scores: -11.98 and 11.98), t(187.73) = 2.33, p = 0.01** |
|  |
|  |
| **The TOST procedure based on Welch's t-test indicated that the observed effect size (d = -0.14) was significantly within the equivalent bounds of d = -0.5 and d = 0.5, (or in raw scores: -12.46 and 12.46), t(54.85) = 1.8, p = 0.038** |
|  |
|  |

***Positive Arousal***

The non-significant difference between C#1 (*M* = 30.64, *SD* = 15.51) and C#2 (*M* = 34.70, *SD =* 16.70, *d* = -.25) was inconclusive.

| **The TOST procedure based on Welch's t-test indicated that the observed effect size (d = -0.25) was not significantly within the equivalent bounds of d = -0.5 and d = 0.5, (or in raw scores: -8.06 and 8.06), t(62.77) = 1.23, p = 0.112** |
| --- |
|  |
|  |

***Experiment 1: Correlations between implicit racial bias and social cognition***

Equivalence tests indicated that the observed effect sizes were statistically equivalent for relationships between race-IAT scores and imitative tendencies, emotion recognition, visual perspective taking, all sub-scales of self-reported trait empathy (EC, PT and PD) and negative arousal measured on the SAE. The relationship between the race-IAT and negative concern was inconclusive.

**Imitative Tendencies:**

| **The TOST procedure indicated that the observed effect size between the IAT and imitative tendencies (r = -0.01) was significantly within the equivalent bounds of r = -0.2 and r = 0.2, p = 0.003** |
| --- |
|  |
|  |

**Emotion Recognition:**

| **The TOST procedure indicated that the observed effect size between the IAT and emotion recognition (r = -0.04) was significantly within the equivalent bounds of r = -0.2 and r = 0.2, p = 0.008** |
| --- |
|  |
|  |

**Self-reported Empathic Concern (EC):**

| **The TOST procedure indicated that the observed effect size between the IAT and self-reported empathic concern (r = -0.01) was significantly within the equivalent bounds of r = -0.2 and r = 0.2, p = 0.002** |
| --- |
|  |
|  |

**Self-reported Perspective Taking (PT)**

| **The TOST procedure indicated that the observed effect size between the IAT and self-reported perspective taking (r = -0.04) was significantly within the equivalent bounds of r = -0.2 and r = 0.2, p = 0.008** |
| --- |
|  |
|  |

**Self-reported Personal Distress (PD):**

| **The TOST procedure indicated that the observed effect size between the IAT and self-reported personal distress (r = -0.03) was significantly within the equivalent bounds of r = -0.2 and r = 0.2, p = 0.004** |
| --- |
|  |
|  |

**Visual Perspective Taking:**

| **The TOST procedure indicated that the observed effect size between the IAT and visual perspective taking (r = 0.07) was significantly within the equivalent bounds of r = -0.2 and r = 0.2, p = 0.025** |
| --- |
|  |
|  |

**Negative Concern:**

| **The TOST procedure indicated that the observed effect size between the IAT and negative concern on the SAE (r = 0.13) was not significantly within the equivalent bounds of r = -0.2 and r = 0.2, p = 0.141** |
| --- |
|  |
|  |

**Negative Arousal:**

| **The TOST procedure indicated that the observed effect size between the IAT and negative arousal on the SAE (r = 0.08) was significantly within the equivalent bounds of r = -0.2 and r = 0.2, p = 0.034** |
| --- |
|  |
|  |

**Experiment 2: Task Performance Checks**

***Implicit Racial Bias***

A one-sample *t*-test indicated that IAT scores differed significantly from zero, with participants displaying a weak pro-White/anti-Black bias (IAT *d, M* = .29, *SD* = .35), *t*(236) = 12.77, *p* < .001, Cohen’s *d* = .83.

***Imitative Tendencies***

A paired-samples *t*-test indicated that RTs were quicker on Compatible (*M* = 546.04, *SD* = 75.21) relative to Incompatible trials on the SRC task (*M* = 557.56, *SD* = 75.72), *t*(213) = -5.35, *p* < .001, *d_z_* = .36.

***Visual Perspective Taking***

A 2 (Perspective: Self vs. Other) x 2 (Consistency: Consistent vs. Inconsistent) repeated-measures Analysis of Variance (ANOVA) indicated a main effect of Perspective, *F*(1, 221) = 18.33, *p* < .001, *η_p_^2^* = .08, and a main effect of Consistency , *F*(1, 221) = 130.41, *p* < .001, *η_p_^2^* = .37 on the Dot task. Participants were quicker to respond accurately on Self (*M* = 805.18, *SD* = 177.16) relative to Other trials (*M* = 825.90, *SD* = 174.78), *p* < .001, *d_z_* = .29, and on Consistent (*M* = 789.76, *SD* = 174.93) compared to Inconsistent trials (*M* = 841.33, *SD* = 176.12), *p* < .001. *d_z_* = .79.

***Affective Empathy***

*Empathic Concern.* A paired-samples *t-*test indicated that empathic concern was greater for negative (*M* = 48.11, *SD* = 7.93) relative to positive images (*M* = 10.92, *SD* = 4.39), *t*(237) = 66.20, *p* < .001, *d_z_* = 4.29.

*Arousal.* A paired-samples *t-*test indicated that arousal was greater for negative (*M* = 28.75, *SD* = 12.10) relative to positive images (*M* = 21.49, *SD* = 9.11), *t*(237) = 7.87 *p* *<* .001, *d_z_* = .51.

**Experiment 2: Confirmatory Analyses**

In our preregistration, we first planned to assess whether our other tasks of social cognition also reveal racial biases by conducting a series of 3 (Participant Race: White, Black, Asian [B-S]) x 2 (Actor Race: White, Black [W-S]) mixed-design ANOVAs on imitative tendencies, emotion recognition, and visual perspective taking, with an additional two-level within-factor of Valence (Positive, Negative [W-S]) for empathic concern and arousal. However, despite advertising this study to the Black, Asian, and Minority Ethnic (BAME) student societies, the final sample size of Black (*n* = 20) and Asian participants was small (*n* = 29), meaning that such analyses would only be able to reliably detect moderate-to-large effects (*d* > .59) with 80% power. The following analyses are therefore not reported in the manuscript and are noted as an explicit deviation to the registered analyses. These analyses are reported here for completeness, but caution should be taken in their interpretation.

***Implicit Racial Bias***

A one-way ANOVA indicated implicit racial bias differed significantly by participant race, *F*(2, 234) = 9.63, *p* < .001, *ηp2* = .08; White participants expressed greater pro-White/anti-Black bias relative to Black participants who expressed neutral implicit attitudes (*p* < .001, *d* = .91). The observed effect size was inconclusive between White and Asian participants (*p* = .09, *d* = .40) and Black and Asian participants (*p* = .24, *d* = .48).

***Imitative Tendencies***

There was no significant main effect of participant race, *F*(2, 211) = .43, *p* = .65, *η_p_^2^* = .004, or actor race *F*(1, 211) = .02, *p* = .90, *η_p_^2^* < .001, nor a significant two-way interaction between participant and actor race, *F*(2, 211) = .19, *p* = .82, *η_p_^2^* = .002. Contrary to predictions, White participants did not demonstrate greater imitative tendencies for White relative to Black actors – the observed effect size (*p* = .50, *d_z_* = .06) fell within the equivalence bounds. However, this was inconclusive for Black (*p* = .81, *d_z_* = .06) and Asian participants’ (*p* = .70, *d_z_* = .06) responses to a White relative to Black actor.

***Emotion Recognition***

There was no significant main effect of participant race, *F*(2, 234) = .69, *p* = .50, *η_p_^2^* = .006, but a significant main effect of actor race, *F*(1, 234) = 5.65, *p* = .018, *η_p_^2^* = .02, indicating that participants were better at recognising the emotions of White relative to Black actors (*p* = .018, *d_z_* = .18). There was no significant two-way interaction between participant and actor race, *F*(2, 234) = .01, *p* = .99, *η_p_^2^* < .001. When evaluating this for equivalence, however, White participants exhibited significantly better emotion recognition for White relative to Black actors (*p* < .01, *d_z_* = .35), but this was inconclusive for Black (*p* = .30, *d_z_* = .33) and Asian participants (*p* = .17, *d_z_* = .32).

***Visual Perspective Taking***

There was no significant main effect of participant race, *F*(2, 219) = 2.57, *p* = .08, *η_p_^2^* = .02, but a significant main effect of actor race^[[3]](#footnote-3)^ *F*(1, 219) = 23.10, *p* < .001, *η_p_^2^* = .10; participants were significantly quicker to take the conflicting perspective of the Black relative to White actor (*p* < .001, *d_z_ =* .43). There was no significant two-way interaction between participant and actor race, *F*(2, 219) = 0.55, *p* = .58, *η_p_^2^* = .005. Against predictions, White participants were quicker to take the perspective of the Black relative to White actor (*p* < .001, *d_z_* = .44). Consistent with predictions, Black participants were quicker to take the perspective of the Black relative to White actor (*p* = .046, *d_z_* = .36). Finally, Asian participants were quicker to take the perspective of the Black relative to White actor (*p* = .01, *d_z_* = .47). As such, all participants were quicker to take the Black relative to White actor’s perspective irrespective of their own racial identity.

***Affective Empathy***

*Empathic Concern*. There was no main effect of participant race, *F*(2, 234) = .01, *p* = .99, *η_p_^2^* < .001, but a significant main effect of actor race, image valence, and a three-way interaction between participant race, actor race and image valence, *F*(2, 234) = 3.78, *p* = .02, *η_p_^2^* = .03. In response to positively valence images, White participants reported greater concern for White relative to Black actors (*p* < .001, *d_z_* = .19), whilst Black participants reported greater concern for Black compared with White actors (*p* = .003, *d_z_* = .34). This was inconclusive for Asian participants (*p* = .43, *d_z_* = .13). Conversely, both White (*p* < .001, *d_z_* = .39) and Asian participants (*p* = .001, *d_z_ =* .42) reported greater concern for Black compared with White actors in negative valence images. However, this was inconclusive for Black participants’ concern for Black relative to White actors (*p* = .08, *d_z_* = .21).

*Arousal*. There was no main effect of participant race, *F*(2, 234) = .63, *p* = .53, *η_p_^2^* < .01, but a significant main effect of actor race, image valence, and a three-way interaction between participant race, actor race and image valence, *F*(2, 234) = 3.62, *p* = .028, *η_p_^2^* = .03. Black participants reported higher arousal towards positive valence images depicting Black relative to White actors (*p* < .001, *d_z_* = .83). However, this was statistically equivalent for White participants’ positive arousal for White and Black actors (*p* = .07, *d_z_* = .19), and inconclusive for Asian participants’ (*p* = .06, *d_z_* = .34). Against predictions, all participants expressed greater arousal for Black relative to White actors in negative valence images (all *p* < .03). Table S1 presents the descriptive statistics.

Table S1. *Descriptive statistics (means [SD]) for all experimental measures as a function of participant and actor race. Abbreviations are provided in Table 2.*

| **Measures** | **Participant Race** | | | | | | | |
| --- | --- | --- | --- | --- | --- | --- | --- | --- |
|  | White | | Black | | Asian | | Total | |
| IAT | .34 (.34) | | .03 (.34) | | .19 (.33) | | .29 (.35) | |
|  | ***Actor Race*** | | | | | | | |
|  | W | B | W | B | W | B | W Total | B Total |
| Imitation  eRec  VPT  Concern*_POS_*  Concern*_NEG_*  Arousal*_POS_*  Arousal*_NEG_* | 13.49 (39.95)  .94  (.07)  875.11 (184.17)  11.21 (6.06)  47.21 (11.13)  20.77 (12.04)  27.99 (16.12) | 10.71 (39.90)  .92  (.08)  822.30 (180.24)  9.91 (6.17)  49.63 (11.44)  21.56 (13.09)  30.02 (17.38) | 6.75 (40.10)  .92  (.07)  970.95 (158.82)  13.50 (6.82)  43.50 (14.25)  22.73 (13.47)  27.45 (14.69) | 3.86 (37.65)  .90  (.08)  910.94 (173.88)  16.14 (10.94)  45.73 (13.53)  28.95 (17.72)  30.09 (16.77) | 10.61 (49.00)  .94  (.06)  923.10 (257.56)  10.71 (4.62)  46.87 (10.89)  19.52 (11.68)  25.84 (16.54) | 14.36 (47.69)  .92  (.06)  841.91 (263.22)  10.13 (5.02)  50.65 (9.02)  21.48 (14.24)  28.94 (19.75) | 12.47 (41.14)  .93  (.07)  889.79 (194.24)  11.35 (5.99)  46.82 (11.42)  20.79 (12.11)  27.66 (16.00) | 10.56 (40.71)  .92 (.08)  832.76 (192.73)  10.51 (6.83)  49.40 (11.38)  22.24 (13.83)  29.88 (17.58) |

**Experiment 2: Exploratory Analyses**

***Imitative Tendencies***

There was no significant difference in imitative tendencies towards an own-race (*M* = 12.45, *SD* = 39.73) relative to other-race actor (*M* = 10.28, *SD* = 39.83), *t*(184) = .59, *p* = .56, with the observed effect size (*d_z_* = -.04) statistically within the equivalence bounds.

***Emotion Recognition***

Participants showed significantly better emotion recognition towards an own-race (*M* = .93, *SD* = .07) relative to other-race actor (*M* = .92, *SD* = .08), *t*(205) = 2.47, *p* = .014, *d_z_* = .18.

***Perspective Taking***

Participants were significantly quicker to judge the perspective of an other-race (*M* = 837.62, *SD* = 183.03) relative to the own-race actor (*M* = 878.80, *SD* = 13.17), *t*(193) = 4.38, *p* < .001, *d_z_* = .31.

***Affective Empathy***

*Empathic Concern.* Participants reported higher positive concern for own-race (*M* = 11.73, *SD* = 6.89) relative to other-race actors (*M* = 10.29, *SD* = 6.33), *p* < .001, *d_z_* = .36. However, they reported higher negative concern towards other-race (*M =* 48.97*, SD =* 11.88) relative to own-race actors (*M* = 47.05, *SD* = 11.38), *t*(205) = -4.71, *p* < .001, *d_z_* = .34.

*Arousal.* Participants reported higher negative arousal towards other-race (*M* = 29.74, *SD* = 17.10) relative to own-race actors (*M* = 28.21, *SD* = 16.16), *t*(205) = -3.79, *p* < .001, *d_z_* = .26. However, there was no significant difference in positive arousal ratings between own- and other-race actors, *t*(205) = -.09, *p* = .93. Equivalence tests indicated that the observed effect size (*d_z_* = .0) was significantly within the equivalence bounds.

1. Samson et al. (2010) found no effects of actor gender on VPT performance. [↑](#footnote-ref-1)
2. Note, *SD* was calculated from the standard error provided by SPSS’s estimates for main effects. [↑](#footnote-ref-2)
3. Although both blocks depicting the Black actor came after those presenting the White actor, we ruled out the possibility that that main effect of actor race was due to an order effect by conducting an exploratory 2 (Actor Race: White, Black) x 2 (Perspective: Consistent, Inconsistent) ANOVA on ‘Other’ perspective trials. This revealed a two-way interaction between actor race and perspective consistency, *F*(1, 220) = 121.79, *p* < .001, *η_p_^2^* = .36, with participants quicker to take the perspective of the Black relative to the White actor when perspectives were inconsistent, but quicker to take the perspective of the White relative to the Black actor when perspectives were consistent (all *p* < .001). [↑](#footnote-ref-3)
